# Genetic map‐guided genome assembly reveals a virulence‐governing minichromosome in the lentil anthracnose pathogen *Colletotrichum lentis*

**DOI:** 10.1111/nph.15369

**Published:** 2018-08-04

**Authors:** Vijai Bhadauria, Ron MacLachlan, Curtis Pozniak, Aurelie Cohen‐Skalie, Li Li, Jerlene Halliday, Sabine Banniza

**Affiliations:** ^1^ Crop Development Centre/Department of Plant Sciences University of Saskatchewan Saskatoon SK S7N 5A8 Canada; ^2^ Swift Current Research and Development Center Agriculture and Agri‐Food Canada Swift Current SK S9H 3X2 Canada

**Keywords:** conditionally dispensable chromosomes, disease resistance, effectors, genomics, genotyping‐by‐whole‐genome shotgun sequencing, legumes, pathogens

## Abstract

- *Colletotrichum lentis* causes anthracnose, which is a serious disease on lentil and can account for up to 70% crop loss. Two pathogenic races, 0 and 1, have been described in the *C. lentis* population from lentil.
- To unravel the genetic control of virulence, an isolate of the virulent race 0 was sequenced at 1481‐fold genomic coverage. The 56.10‐Mb genome assembly consists of 50 scaffolds with *N*_50_ scaffold length of 4.89 Mb. A total of 11 436 protein‐coding gene models was predicted in the genome with 237 coding candidate effectors, 43 secondary metabolite biosynthetic enzymes and 229 carbohydrate‐active enzymes (CAZymes), suggesting a contraction of the virulence gene repertoire in *C. lentis*.
- Scaffolds were assigned to 10 core and two minichromosomes using a population (race 0 × race 1, *n *=* *94 progeny isolates) sequencing‐based, high‐density (14 312 single nucleotide polymorphisms) genetic map. Composite interval mapping revealed a single quantitative trait locus (QTL), *qClVIR‐11*, located on minichromosome 11, explaining 85% of the variability in virulence of the *C. lentis* population. The QTL covers a physical distance of 0.84 Mb with 98 genes, including seven candidate effector and two secondary metabolite genes.
- Taken together, the study provides genetic and physical evidence for the existence of a minichromosome controlling the *C. lentis* virulence on lentil.

## Introduction

*Colletotrichum lentis* Damm can infect various legume species, such as lentil (*Lens culinaris*), faba bean (*Vicia faba*), narrow‐leaf vetch (*Vicia americana*), field pea (*Pisum sativum*) and, under certain conditions, chickpea (*Cicer arietinum*) (Gossen *et al*., 2009). It is a very serious and damaging pathogen on cultivated lentil (*Lens culinaris* Medik. ssp. *culinaris*) in western Canada during warm and humid growing seasons, and can account for up to 70% crop loss (Morrall & Pedersen, 1991; Chongo *et al*., 1999), resulting in economic losses higher than those for most other pathogens of grain legumes.

**Figure 1:**
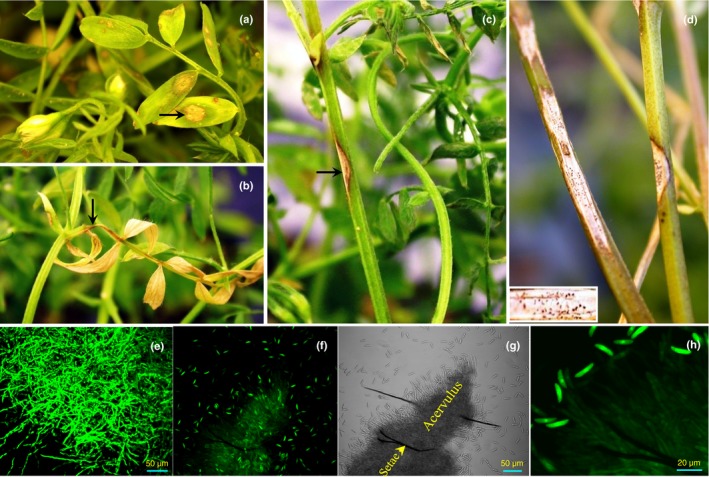
Symptoms of anthracnose on lentil (*Lens culinaris* ssp. *culinaris*). Anthracnose lesions (arrows) on (a) leaflet, (b) petiole and (c) stem; (d) microsclerotia (pin‐sized dark brown to black structures in lesion) of the causal pathogen *Colletotrichum lentis* on lentil stem; (e) crushed microsclerotium; and (f–h) conidial dispersal.

*Colletotrichum lentis* is a foliar pathogen and infects all aboveground parts of lentil, including leaflets, petioles and stems (Fig. 1a–c). The fungus can survive for up to 5 yr in the soil as microsclerotia (Fig. 1d,e) on lentil stubble and debris left in the field after harvest, which serve as the primary source of inoculum for subsequent lentil crops (Buchwaldt *et al*., 1996). The pathogen exploits a biphasic intracellular hemibiotrophic infection strategy to colonize lentil plants. The infection cycle starts when asexual spores (conidia) of the pathogen land on the host surface and germinate to form round infection structures called appressoria. A narrow penetration peg emerging from the base of the melanized appressorium ruptures the plant surface to form a spherical infection vesicle, which initiates the symptomless phase of infection, characterized by a thick biotrophic primary hypha emanating from the infection vesicle. Unlike other biotrophic and hemibiotrophic pathogens, such as *Ustilago maydis*,* Blumeria graminis* and *Magnaporthe oryzae*,* C. lentis* forms a weak plant–pathogen interphase characterized by a swift pulling away of the lentil plasma membrane from the biotrophic primary hypha during plasmolysis, indicating that the pathogen employs a unique infection strategy during the quiescent period of infection (Bhadauria *et al*., 2011). The length of the biotrophic phase varies and can last up to several days depending on temperature and humidity (Armstrong‐Cho *et al*., 2012). The biotrophic phase ceases once the pathogen deploys the effector CtNUDIX, which probably dismantles the integrity of the lentil plasma membrane (Bhadauria *et al*., 2013a,b). Disease symptoms appear with the onset of the necrotrophic phase, characterized by thin secondary hyphae, which kill and macerate plant tissues potentially through the massive production of ToxB and hydrolytic enzymes, as manifested by initially water‐soaked lesions that turn necrotic (Bhadauria *et al*., 2011, 2015). Eventually, orange‐ to salmon‐colored, single‐celled conidia ooze out of subcuticular fruiting structures (acervuli; Fig. 1f–h) in mature lesions, and are dispersed by rain splash to infect healthy neighboring plants, thus serving as a secondary source of inoculum. Under favorable conditions, the latent period of *C. lentis* infection is 7 d, allowing the pathogen to complete multiple cycles of infection during the cropping season (Chongo & Bernier, 2000).

Isolates of *C. lentis* from lentil have been grouped into two pathogenic races, originally named Ct0 and Ct1 (Buchwaldt *et al*., 2004), but later renamed simply race 0 and 1 to avoid confusion with host resistance or pathogen virulence gene designation (Armstrong‐Cho *et al*., 2012), a renaming which has since become more logical considering the reclassification of this pathogen from *C. truncatum* to the new species *C. lentis* (Damm *et al*., 2014). Races of *C. lentis* lack the characteristic features of the classical physiological races, such as completely resistant host cultivars and clearly incompatible reactions characterized by the hypersensitive response. Although differences in responses to the races in partially resistant and fully susceptible lentil cultivars are distinct, there is a strong quantitative effect involved in the interactions (Armstrong‐Cho *et al*., 2012). Sources of partial resistance against the less virulent race 1 have been identified in *L. culinaris* and were successfully introgressed into lentil cultivars, such as *L. culinaris* spp. *culinaris* cv CDC Robin (Vandenberg *et al*., 2002). However, high levels of resistance to the virulent race 0 have remained elusive in the cultivated gene pool, but have been identified in the tertiary gene pool of *Lens*, specifically in *L. ervoides* (Tullu *et al*., 2006; Fiala *et al*., 2009; Bhadauria *et al*., 2017a).

Several draft and optical map‐based genomes of species in the genus *Colletotrichum* have been published in past years, addressing and comparing the molecular mechanisms governing the pathogenic life of the species (O'Connell *et al*., 2012; Baroncelli *et al*., 2014; Crouch *et al*., 2014; Gan *et al*., 2016; Zampounis *et al*., 2016; Buiate *et al*., 2017). Although an increasingly more comprehensive understanding of the pathogenesis of these pathogens is thus emerging, the key genes determining compatibility and virulence in the host have not been identified, with the exception of the *C. lindemuthianum*–*Phaseolus vulgaris* pathosystem, which follows a gene‐for‐gene concept as described by Flor in 1946 (Luna‐Martínez *et al*., 2007). With the inception of next‐generation sequencing, especially massively parallel sequencing, it has become feasible to dissect fungal genomes for loci associated with virulence, in particular when ascospore‐derived populations from crosses are available, as is the case for *C. lentis*.

In this study, we sequenced, assembled and annotated the genome of the virulent *C. lentis* isolate CT‐30 (race 0). A high‐density genetic map (14 312 single nucleotide polymorphism (SNP) variants) with 12 linkage groups (LGs) was generated through genotyping‐by‐whole‐genome shotgun sequencing of an ascospore‐derived population (*n* = 94) from the cross of CT‐30 (race 0) and CT‐21 (race 1). The genetic map aided in the ordering and orientation of the assembly scaffolds into pseudomolecules/chromosomes. Using SNPs and virulence data of the population on race 1‐resistant and race 0‐susceptible CDC Robin, a quantitative trait locus (QTL) controlling virulence in *C. lentis* was mapped onto a minichromosome. The data obtained in the study provide genetic and physical evidence of 10 core and two minichromosomes, and indicate that *C. lentis* regulates its virulence on lentil through a minichromosome.

## Materials and Methods

### Fungal isolates

The fertile isolates CT‐30 (race 0) and CT‐21 (race 1) were used as parental isolates to develop an ascospore‐derived progeny isolate population. The virulent isolate CT‐30 was used as a reference isolate to generate a *de novo* draft genome assembly of *C. lentis*. All isolates were routinely maintained on oat meal agar (OMA: 30 g blended quick oats (The Quaker Oats Company, Chicago, IL, USA), 8.8 g agar (Becton, Dickinson and Company, Sparks, MD, USA), 1 l deionized water) plates supplemented with 0.01% chloramphenicol (AMRESCO, Solon, OH, USA).

### Genome sequencing and assembly

Genomic DNA (gDNA) from mycelia of isolate CT‐30 was extracted using the DNeasy Plant Mini Kit (Qiagen Inc., Hilden, Germany). The CT‐30 gDNA was sequenced on the HiSeq 2000 and MiSeq sequencers (Illumina Inc., San Diego, CA, USA). Three paired‐end libraries with insert sizes of 250, 300 and 350 bp were constructed using the TruSeq DNA Library Prep Kit (Illumina Inc.) and sequenced to generate 2 × 100‐bp and 2 × 250‐bp reads. In addition, two mate‐pair libraries with insert sizes of 5 and 10 kb were prepared using the Nextera DNA Library Prep Kit (Illumina Inc.) and sequenced on the HiSeq 2000 platform to generate 2 × 100‐bp reads. The sequencing was performed at the Centre d'Innovation Génome Québec, Montréal, Canada. Duplicates, singletons, low‐quality reads (< Q30) and adapter sequences were removed using Trimmomatic (Bolger *et al*., 2014). High‐quality HiSeq and MiSeq paired‐end reads were assembled into contigs using SOAPdenovo2 (Luo *et al*., 2012). The contigs were then anchored with a kmer of 81 using the mate‐pair reads.

This Whole Genome Shotgun project has been deposited at DDBJ/ENA/GenBank under accession NWBT00000000. The version described in this article is version NWBT01000000.

### Genome annotation

Before genome annotation, repeats from assembly scaffolds were masked using repeatmasker v.4.0.5 (Smit *et al*., 1996) and a fungal‐specific library of repetitive elements from RepBase (Jurka *et al*., 2005). The masked genome was used for *ab initio* gene model prediction using fgenesh (Solovyev *et al*., 2006) with *Colletotrichum* species‐specific (*C. fioriniae* Marcelino & Gouli *ex* R.G. Shivas & Y.P. Tan, *C. higginsianum* Sacc., *C. graminicola* (Ces.) G.W. Wilson, *C. orbiculare* Damm, P.F. Cannon & Crous and *C. gloeosporioides* (Penz.) Penz. & Sacc.) gene‐finding parameters.

Gene space coverage and genome completeness were determined using busco with a set of 1438 fungi‐specific conserved orthologous genes (Simão *et al*., 2015). Non‐coding RNA species, such as tRNA and rRNA, were predicted using tRNAscan‐SE (Lowe & Eddy, 1997) and rnammer (Lagesen *et al*., 2007), respectively. smurf (Khaldi *et al*., 2010) and dbCAN (Yin *et al*., 2012) were used to predict secondary metabolite cluster genes and carbohydrate‐active enzymes (CAZymes), respectively, in the *C. lentis* genome. Candidate effectors were identified following a pipeline described previously (Bhadauria *et al*., 2015, 2017b). signalP v.4.0 (Petersen *et al*., 2011) and protcomp‐AN v.10.0 (Softberry Inc., Mount Kisco, NY, USA), and tmhmm (Sonnhammer *et al*., 1998), were used to predict subcellular localization and transmembrane helices in the *C. lentis* proteome, respectively. predgpi (Pierleoni *et al*., 2008), netnglyc v.1.0 and netoglyc v.4.0 (Steentoft *et al*., 2013) were used to scan the *C. lentis* proteome for glycosylphosphatidylinositol (GPI) anchor addition sites, *N*‐glycosylation sites and *O*‐glycosylation sites, respectively.

### Development of a biparental *C. lentis* population

A biparental ascospore‐derived *C. lentis* population was generated *in vitro* by mating race 0 isolate CT‐30 with race 1 isolate CT‐21 following the procedure developed by Armstrong‐Cho & Banniza (2006). In short, stems of senescent plants of lentil (*Lens culinaris* Medik.) cultivar Eston were cut into 5‐cm pieces and autoclaved. The stems were then soaked in a 1 : 1 conidial suspension (2 × 10^5^ conidia ml^−1^) of CT‐30 and CT‐21 in 20‐ml test tubes and incubated at 22°C with gentle shaking for 2 h. The stems were removed and placed in Petri dishes containing filter paper placed on top of 1.5% water agar. The plates were sealed with parafilm (Bemis Co. Inc., Neenah, WI, USA) and incubated at room temperature in the dark for 10–14 d for the induction of sexual fruiting structures (perithecia).

Mature perithecia were removed from the stems and washed individually in several droplets of sterile water to remove conidia. Each perithecium was crushed on a glass slide to release asci, which were then transferred into Petri dishes with OMA. Asci were teased gently to release and separate the ascospores before being incubated at 22°C until the ascospores had germinated. Individual germinated ascospores were transferred to new Petri dishes with OMA.

### Phenotyping of the ascospore‐derived *C. lentis* population for virulence

Ninety‐four arbitrarily selected ascospore‐derived isolates, CT‐21 and CT‐30 were grown on OMA medium in Petri dishes for 10 d; conidia were harvested and spore suspensions were prepared with 50 000 spores ml^−1^.

Isolates were phenotyped in a phytotron chamber using an incomplete block design with six replications. Differential lentil cultivar CDC Robin (partially resistant to race 1 and susceptible to race 0) and susceptible cultivar Eston were grown at four plants per 10 × 10‐cm^2^ square pot filled with a 3 : 1 mix of Sunshine Professional Mix no. 4 (Sun Gro Horticulture, Bellevue, WA, USA) and perlite (Special Vermiculite Canada, Winnipeg, MB, Canada) for 3 wk. For inoculation, each plant was sprayed with 3 ml of conidial suspension and pots were incubated in a mist tent with 100% relative humidity for 24 h. Disease severity on leaves and stems was assessed separately at 7 d post‐inoculation (7 dpi) using the Horsfall–Barratt scale (Horsfall & Barratt, 1945). As anthracnose severity on stems provides the clearest differentiation between the races, only data from stem lesions are presented.

Disease scores were transformed into mid percentage values and averaged for each pot before analysis of variance using the mixed model procedure (proc mixed) of Sas v.9.3 (SAS Institute, Cary, NC, USA). Isolates and lentil cultivar were considered as fixed factors, whereas replicates and incomplete blocks were considered as random factors. The Shapiro–Wilk test implemented in proc univariate and mixtools implemented in R were used to determine the distribution of anthracnose severity on lentil stems.

### Whole‐genome shotgun sequencing and variant calling

gDNA was extracted from mycelia of ascospore‐derived isolates and parental isolates CT‐30 and CT‐21 using the DNeasy Plant Mini Kit (Qiagen Inc.). Ninety‐six gDNA libraries were prepared using an Illumina TruSeq DNA LT Sample Prep Kit (Illumina Inc.) following the manufacturer's instructions. In short, 1 μg of high‐quality gDNA (A260/280, 1.8–2.0; A260/230, 2.0–2.4) per isolate was sheared mechanically, and the resulting fragments were end‐repaired and three prime adenylated. Paired‐end barcoded adapters were ligated to the A‐tailed DNA fragments. Libraries were multiplexed into four pools, 24 libraries per pool. Gel‐based size selection was performed at this stage, and 300‐bp DNA inserts were purified from gel bands. Library quality and size were checked on a BioAnalyzer 2100 (Agilent Technologies Inc., Wilmington, DE, USA) using the Agilent 1000 DNA chip (Agilent Technologies Inc., Palo Alto, CA, USA). The pooled libraries were quantified using the KAPA library quantification kit for Illumina sequencing platforms (KAPA Biosystems Inc., Woburn, MA, USA). The adapter dimer contamination‐free, 20‐pM, 24‐plex library was sequenced on an Illumina HiSeq 2000 platform to generate 2 × 100‐bp reads at the Centre d'Innovation Génome Québec, Montréal, Canada.

High‐quality paired‐end reads (Q > 30 with a sliding window of four bases) were mapped onto the *C. lentis* genome assembly using novalign (http://novocraft.com/). SNP variants were called from the mapped reads where parental isolates showed polymorphisms and were recorded in variant call format (vcf) using samtools mpileup (Li *et al*., 2009). In total, 96 vcf files were generated and merged into a population vcf before linkage mapping of SNPs. Post‐mapping quality filtering was performed as described by Bhadauria *et al*. (2017a).

### Genetic linkage mapping, QTL detection and comparative mapping

The CT‐30 × CT‐21 genetic map was constructed by grouping high‐quality SNP markers into LGs using madmapper Python scripts (http://cgpdb.ucdavis.edu/XLinkage/MadMapper) and ordering markers within the groups using the record algorithm implemented in the Windows version of record (Van Os *et al*., 2005). checkmatrix Python script (Kozik & Michelmore, 2006) was used to validate the genetic map. Composite interval mapping (CIM) implemented in the Windows version of QTL cartographer v.2.5 (Wang *et al*., 2012) was used to map the QTL controlling the *C. lentis* virulence on lentil. A logarithm of the odds (LOD) score threshold of three was used to locate the QTL.

Homologous pseudomolecule sequences between *C. lentis* and *C. higginsianum* were identified using nucmer and visualized using mummerplot of mummer software (Kurtz *et al*., 2004).

### RNA‐sequencing (RNA‐Seq) and reverse transcription‐quantitative polymerase chain reaction (RT‐qPCR)

We used transcriptome data obtained from CT‐30‐infected tissues of lentil cultivar Eston displaying three fungal infection stages (appressoria‐assisted penetration of epidermal cells (23 h post‐inoculation (hpi)), biotrophic hyphae (55 hpi) and necrotrophic hyphae (96 hpi)) and vegetative mycelia primarily to assist in and consolidate the annotation (gene model prediction) of the *C. lentis* genome. We used the cultivar Eston instead of the differential CDC Robin as it is fully susceptible to both races, and hence can capture expressed gene models during various stages of the *C. lentis* infection process. As the experimental conditions were replicated (three biological replications), we also used the data to profile differential gene regulation.

Spray inoculation was conducted as described above in a randomized complete block design with three replications, and infected lentil tissues were harvested for the three fungal infection stages. Vegetative mycelia were collected as described by Bhadauria *et al*. (2017b). All tissues were flash‐frozen in liquid N_2_ before storing at −80°C. Frozen tissues were ground into a fine powder for RNA extraction. Total RNA was extracted using the SV Total RNA Isolation System (Promega Corp., Fitchburg, WI, USA) following the manufacturer's instructions. In addition, in‐column DNase treatment was also performed during the RNA extraction. RNA samples were quantified on a NanoDrop ND 1000 (NanoDrop Technologies, Wilmington, DE, USA), and samples with an A260 : 280 ratio of 1.8–2.0 and an A260 : 230 ratio of 2.0–2.4 were further assessed for RNA integrity on a BioAnalyzer 2100 (Agilent Technologies) using an Agilent RNA 6000 Nano chip. Samples with an RNA Integrity Number of larger than 7 were used to generate mRNA‐Seq stranded libraries. A total of 500 ng was employed to generate RNA‐Seq libraries using the NEBNext Ultra Directional (stranded) RNA Library Prep kit for Illumina sequencing (New England BioLabs Inc., Ipswich, MA, USA). Libraries were quantified using the KAPA library quantification kit for Illumina sequencing platforms (KAPA Biosystems Inc.). Nine pM of stranded mRNA library was sequenced on an Illumina HiSeq 2500 to generate 125‐bp paired‐end reads. In total, three lanes of the flow cell were used for sequencing.

A *de novo* transcriptome assembly was constructed from high‐quality paired‐end reads following a pipeline developed by Haas *et al*. (2013). The pipeline is based on the trinity assembler suite (Grabherr *et al*., 2011). The differential gene expression analysis was performed using deseq and edger R Bioconductor packages (Anders & Huber, 2010; Robinson *et al*., 2010). Details on RNA‐Seq data analysis can be found in Supporting Information Notes S1.

We used the differential cultivar CDC Robin to analyze the comparative expression of key genes located in the QTL controlling the *C. lentis* virulence on lentil. Genes were functionally annotated through blastx search of peptide sequences in the GenBank non‐redundant (nr) protein database, and by searching for homologous transcripts using the Reference RNA sequence (refseq_RNA) database to identify those with potential involvement in pathogenicity and/or virulence based on other *Colletotrichum* spp. The annotation ‘*C. lentis*‐specific’ was assigned to hits in which functional domains were unknown or unidentified. Selected genes were cross‐referenced with RNA‐Seq data and only differentially selected genes were selected. Infected leaf samples were collected at two stages, biotrophic phase and necrotrophic phase, and RNA was extracted from these samples as described above. The 2^−ΔΔCt^ method (Livak & Schmittgen, 2001) was used to profile the expression of the genes using *C. lentis* glyceraldehyde 3‐phosphate dehydrogenase (GAPDH) and 60S as reference genes and vegetative hyphae as a calibrator.

## Results and Discussion

### High‐quality *C. lentis* genome assembly

Sequencing of the fertile *C. lentis* isolate CT‐30 (race 0) on Illumina HiSeq and MiSeq platforms yielded a genome assembly of 56.10 Mb with a 1481‐fold coverage (Tables 1, S1). A total of 2980 contigs was assembled from 100‐ and 250‐bp paired‐end reads originating from short paired‐end libraries (250, 300 and 350 bp). Anchoring of contigs using 100‐bp paired‐end reads from long mate‐pair libraries (5 and 10 kb) resulted in 50 scaffolds with an *N*_50_ length of 4.89 Mb, which is higher than that of almost all other sequenced *Colletotrichum* species, such as an *N*_50_ scaffold length of 579.19 kb for *C. graminicola* (57.44 Mb, 653 scaffolds), 428.89 kb for *C. orbiculare* (88.3 Mb, 525 scaffolds), 112.81 kb for *C. gloeosporioides* (55.6 Mb, 1241 scaffolds), 137.25 kb for *C. fioriniae* (49.01 Mb, 1108 scaffolds) and 292 kb for *C. incanum* (53.25 Mb, 1036 scaffolds) (O'Connell *et al*., 2012; Gan *et al*., 2013, 2016; Baroncelli *et al*., 2014). The first genome assembly of *C. higginsianum* (53.35 Mb, 367 scaffolds) also had a lower *N*_50_ length of 265.5 kb (O'Connell *et al*., 2012), but, in a recently reported improved version of the *C. higginsianum* genome assembly with 25 scaffolds, the *N*_50_ scaffold length was increased to 5.20 Mb (Zampounis *et al*., 2016). Furthermore, using PacBio and optical mapping, the authors sequenced 11 of the 12 chromosomes from telomere to telomere. Eighteen scaffolds of *C. lentis* contain at least one telomere, as evident by the presence of tandem repeats (TTAGGG)_*n*_ or (CCCTAA)_*n*_. Among these, three (scaffold10, scaffold14 and scaffold16) have both telomeres and hence are full chromosomes. Scaffold14 is a 1.52‐Mb minichromosome.

**Table 1:** The *Colletotrichum lentis* genome assembly features

Genome features	Statistics
Assembly size	56.10 Mb
Contigs	2980
Contig N/L50	248/51.16 kb
Scaffolds	50
Scaffold N/L50	5/4.89 Mb
Gap	1.70%
Protein coding gene models	11 436
GC content	48.62%
tRNA	329
rRNA	54
Repeat elements	4.56%
Gene model coverage^a^	99.58%

a Complete/partial gene models as evaluated by BUSCO (Simão *et al*., 2015).

Nearly 90% (51.0 Mb) of the *C. lentis* genome assembly was contained in 15 scaffolds of varying lengths ranging from 1.46 to 6.69 Mb, suggesting that the *C. lentis* genome assembly is a high‐quality reference assembly and close to the pseudomolecule level. The genome size of *C. lentis* (56.10 Mb) is similar to that of other *Colletotrichum* species, except for *C. orbiculare* whose genome is expanded to 88.3 Mb. A total of 1432 (98.58%) of 1438 benchmarking universal single‐copy fungal orthologous genes (Simão *et al*., 2015) were mapped onto the genome, providing further evidence for a near‐complete genome assembly. Repetitive DNA represents 4.56% of the genome assembly, which is greater than that reported in *C. higginsianum* (1.2%) and *C. gloeosporioides* (0.75%), but less than that in *C. graminicola* (12.2%) and *C. orbiculare* (8.3%). The *C. lentis* genome has a GC content of 48.62%, which is comparable with that of other *Colletotrichum* species.

### Genome annotation reveals reduction in gene models involved in pathogenesis

A total of 11 436 protein‐coding gene models was predicted in the genome of *C. lentis* (Tables 1, S2), lower than that reported for other sequenced *Colletotrichum* species, such as *C. graminicola* (12 006), *C. orbiculare* (13 479), *C. fioriniae* (13 759), *C. higginsianum* (14 651) and *C. gloeosporioides* (15 469). The number of gene models in *C. lentis* is comparable with that of *C. incanum*, whose genome contains 11 852 gene models (Gan *et al*., 2016).

Similarly, with 237 effectors (Table S3), the effectorome of *C. lentis* is reduced relative to that of other *Colletotrichum* species, such as *C. orbiculare* (700) and *C. gloeosporioides* (755) (Gan *et al*., 2016). Twenty‐three of them are unique to *C. lentis*, whereas the remaining have homologs within the genus *Colletotrichum*. Of the 237 candidate effectors, 143 were differentially expressed (false discovery rate (FDR) < 0.05) in the host lentil cv Eston (susceptible to both races) during infection when compared with vegetative mycelia. Nearly 57% of the candidate effectors (135) have known homologs, suggesting a conserved infection strategy across species belonging to the genus *Colletotrichum*. These include various classes of extracellular (EC) proteins known to sequester fungal chitin to evade detection by the host, host‐range‐determining pea pathogenicity protein 1 (Pep1), biotrophy–necrotrophy switch regulator NUDIX, biotrophy‐associated secreted protein 2, cholera enterotoxin subunit A2, cell death‐inducing necrosis and ethylene‐inducing protein 1‐like protein 4, chitin‐binding proteins and CFEM (Common in Fungal Extracellular Membrane proteins) domain‐containing protein.

**Figure 2:**
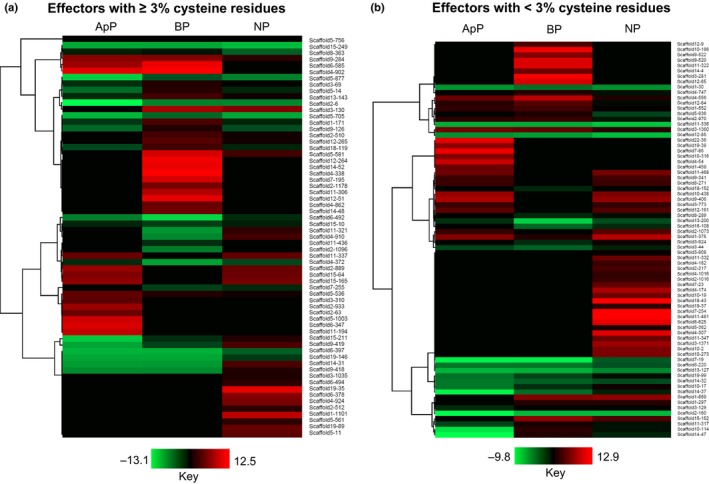
Heatmap representing the expression of candidate effectors (log_2_ fold change (log_2_ FC) ≥ 2, false discovery rate (FDR) < 0.05) of *Colletotrichum lentis in planta* (lentil cultivar Eston) compared with *in vitro*‐grown mycelia. (a) Cysteine‐rich (≥ 3% cysteine residues) candidate effectors; (b) candidate effectors containing < 3% cysteine residues. Hierarchical clustering was performed to cluster gene expression using the one minus Pearson correlation. ApP, appressorium‐assisted penetration (23 h post‐inoculation (hpi)); BP, biotrophic phase (55 hpi); NP, necrotrophic phase (96 hpi).

The number of cysteine‐rich effectors (3.0% or more cysteine residues) was also considerably lower in *C. lentis* (104) compared with *C. orbiculare* (372) and *C. gloeosporioides* (355) (Table S3). Of these, 14 were highly induced during the penetration of lentil cells via appressoria. These include EC7 (scaffold6‐347, 9.6‐fold), EC2 (scaffold5‐1003, 9.2‐fold), EC14 (scaffold11‐194, 8.6‐fold), EC20 (scaffold2‐63, 4.8‐fold), a chitin‐binding protein (scaffold2‐933, 6.8‐fold) and a CFEM domain‐containing protein (scaffold5‐536, 4.3‐fold) (Fig. 2a; Table S4). Nineteen were upregulated during the biotrophic phase wherein lentil epidermal cells were colonized by thick primary hyphae. Among them were EC34 (scaffold4‐338, 11.5‐fold), an alcohol dehydrogenase (scaffold12‐265, 3.9‐fold) and four *C. lentis*‐specific candidate effectors (scaffold14‐52 (10.4‐fold), scaffold14‐48 (5.0‐fold), scaffold13‐143 (3.0‐fold) and scaffold2‐510 (3.0‐fold)). Seventeen candidate effectors were induced during the necrotrophic phase wherein lentil cells were macerated by the secondary hyphae, including two cell wall‐degrading pectate lyases (scaffold19‐35 (10.2‐fold) and scaffold1‐1101 (8.3‐fold)) and a glycoside hydrolase (scaffold6‐378, 5.9‐fold).

Of the 237 candidate effectors, 133 contained 0–2.9% cysteine residues (Table S3). Of these, 19 were induced during the penetration phase via appressoria, including appressorium‐specific protein Cas1 (scaffold7‐86, 11.0‐fold), a cell wall protein (scaffold4‐54, 9.1‐fold), polysaccharide deacetylase (scaffold1‐459, 4.9‐fold), SCP‐like EC protein (scaffold12‐191, 4.3‐fold) and pathogenesis‐associated protein Cap20 (scaffold3‐773, 2.7‐fold) (Fig. 2b; Table S5). Fifteen candidate effectors were upregulated during the biotrophic phase, including three paralogs of the NUDIX effector (scaffold12‐65 (9.7‐fold), scaffold12‐9 (3.4‐fold) and scaffold1‐552 (2.8‐fold)) and EC32 (scaffold11‐322, 10.0‐fold). Thirty candidate effectors were induced during the biotrophic phase, and the majority were involved in plant cell tissue maceration, such as endoglucanase II (scaffold7‐254 (12.9‐fold), scaffold18‐43 (11.8‐fold), scaffold4‐174 (7.9‐fold), scaffold10‐438 (5.7‐fold) and scaffold2‐1016 (2.5‐fold)), endo‐1,4‐β‐xylanase (scaffold6‐625, 10.5‐fold), pectinesterase (scaffold4‐307, 10.2‐fold) and amidohydrolase (scaffold4‐307, 7.2‐fold).

**Figure 3:**
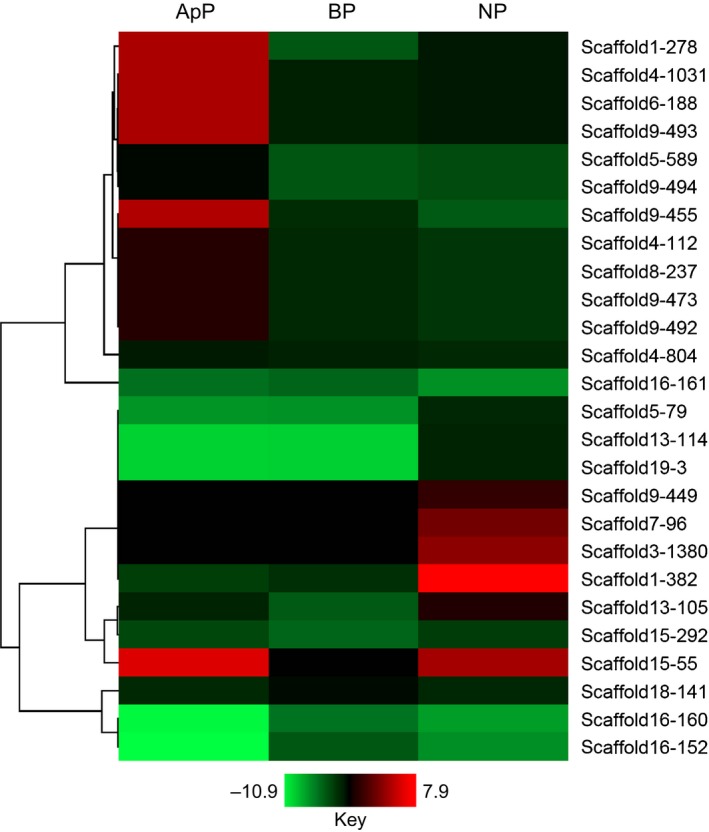
Heatmap representing the expression of secondary metabolite backbone genes (log_2_ fold change (log_2_ FC) ≥ 2, false discovery rate (FDR) < 0.05) of *Colletotrichum lentis in planta* (lentil cultivar Eston) compared with *in vitro*‐grown mycelia. Hierarchical clustering was performed to cluster gene expression using the one minus Pearson correlation. ApP, appressorium‐assisted penetration (23 h post‐inoculation (hpi)); BP, biotrophic phase (55 hpi); NP, necrotrophic phase (96 hpi).

The *C. lentis* genome contains 43 secondary metabolite backbone‐forming proteins, which is lower than the numbers identified in the genomes of *C. higginsianum* (84), *C. graminicola* (53), *C. orbiculare* (54) and *C. gloeosporioides* (76) (Fig. S1). Six were induced during the penetration phase via appressoria, comprising five polyketide synthases (PKSs; scaffold15‐55 (6.1‐fold), scaffold4‐1031 (4.8‐fold), scaffold6‐188 (4.8‐fold), scaffold9‐493 (4.8‐fold) and scaffold1‐278 (4.8‐fold)) and one dimethylallyl tryptophan synthase (scaffold9‐455, 4.9‐fold) (Fig. 3; Tables S6, S7). The induction of *PKS* genes during this phase is consistent with their involvement in appressorium‐dependent penetration as they encode enzymes implicated in melanin biosynthesis. Lack of induction in the expression of backbone genes was evident during the biotrophic phase, whereas four were upregulated during the necrotrophic phase, for example, one non‐ribosomal peptide synthetase (NRPS; scaffold1‐382, 7.9‐fold), two NRPS‐like (male sterility proteins; scaffold3‐1380 (4.0‐fold) and scaffold7‐96 (3.3‐fold)) and one PKS (scaffold15‐55, 4.6‐fold).

**Figure 4:**
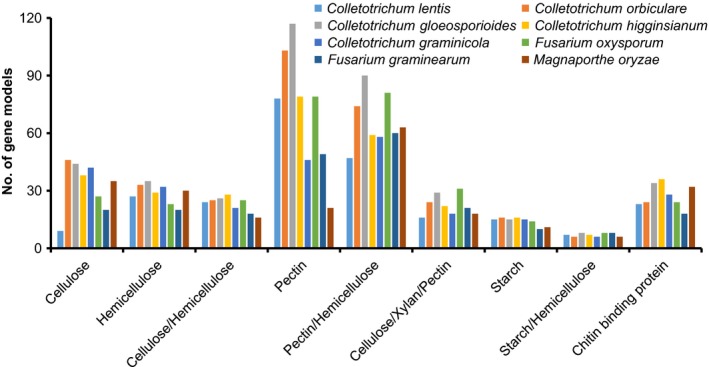
Comparative analysis of carbohydrate‐active enzymes (CAZymes) of *Colletotrichum lentis*. The *y*‐axis indicates the number of gene models encoding CAZymes associated with the degradation of plant cell wall components (*x*‐axis).

**Figure 5:**
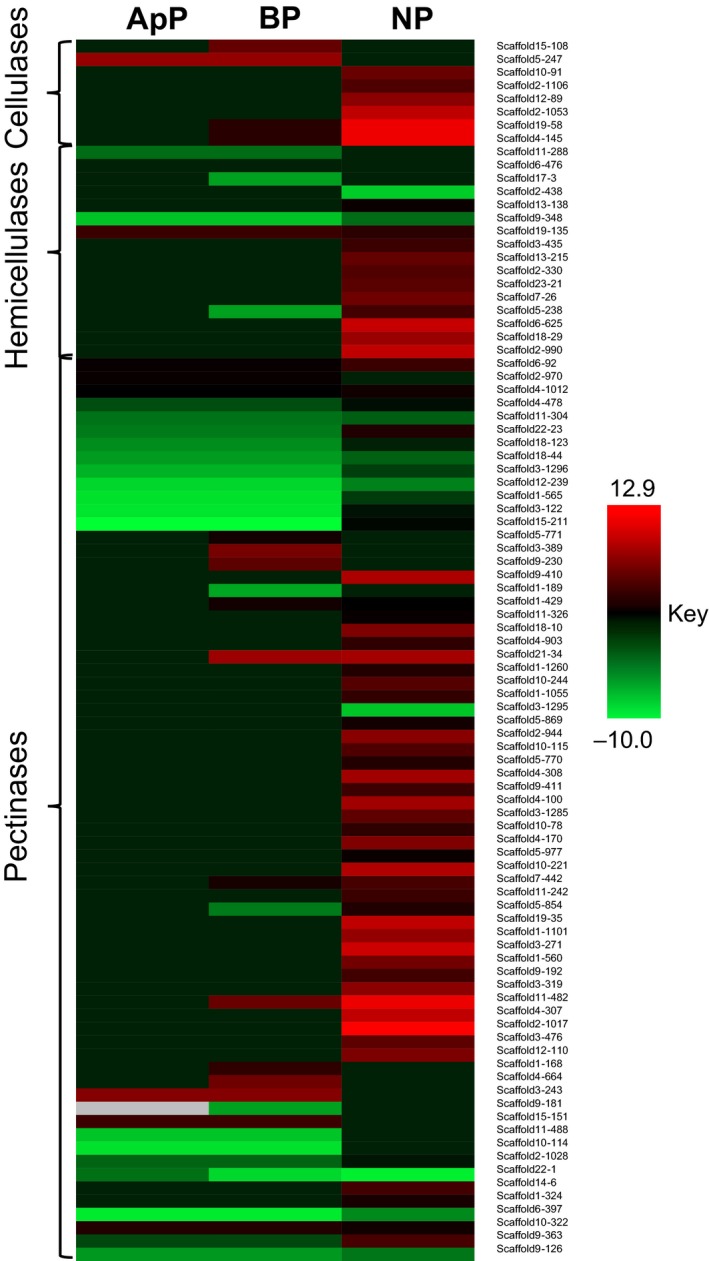
Heatmap representing the expression of *Colletotrichum lentis* genes encoding enzymes required for the degradation of plant cell wall components, such as cellulose, hemicellulose and pectin (log_2_ fold change (log_2_ FC) ≥ 2, false discovery rate (FDR) < 0.05) *in planta* (lentil cultivar Eston) compared with *in vitro*‐grown mycelia. Hierarchical clustering was performed to cluster gene expression using the one minus Pearson correlation. ApP, appressorium‐assisted penetration (23 h post‐inoculation (hpi)); BP, biotrophic phase (55 hpi); NP, necrotrophic phase (96 hpi).

Fungal pathogens deploy CAZymes to gain access to plant tissues by breaking down plant cell wall components, such as cellulose, hemicellulose and pectins. Similar to effectors and secondary metabolites, genome analysis revealed that *C. lentis* encoded fewer CAZymes (229) than any other sequenced genome in the genus, including *C. orbiculare* (327), *C. gloeosporioides* (364), *C. higginsianum* (278) and *C. graminicola* (288) (Fig. 4; Table S8). The contraction of the CAZyme arsenal is attributed to a reduction in cellulose‐degrading enzymes (7), which is four‐ to five‐fold lower than in any other *Colletotrichum* species. Of these 229 CAZymes, 155 were differentially expressed *in planta* when compared with vegetative mycelia (Table S9). Six of the eight cellulases were highly induced during the necrotrophic phase (scaffold2‐1106 (glucanase, 5.1‐fold), scaffold19‐58 (glycosyl hydrolase, 12.3‐fold), scaffold4‐145 (glucanase, 12.3‐fold), scaffold2‐1053 (exoglucanase, 10.2‐fold), scaffold12‐89 (glucanase, 7.8‐fold) and scaffold10‐91 (β‐glucosidase, 6.3‐fold)) (Table S9). Similarly, hemicelluloses (48%) and pectinases (47%) were activated during this phase, including xylanase (scaffold6‐625 (10.5‐fold) and scaffold2‐990 (10.2‐fold)), polysaccharide lyase (12.9‐fold), α‐l‐rhamnosidase (12.2‐fold), cell wall glycosyl hydrolase (10.7‐fold), pectinesterase (10.2‐fold) and pectate lyase (10.2‐fold) (Fig. 5). Some of the CAZymes are also involved in fungal cell wall remodeling or modification to adapt to the *in planta* infection life style. These include those binding fungal chitin to evade recognition by the host defense system, such as intracellular hyphae protein 1 (scaffold3‐243, 7.6‐fold), chitin recognition proteins (scaffold15‐151 (4.3‐fold) and scaffold10‐322 (3.1‐fold)) and chitin deacetylase (scaffold4‐664, 6.6‐fold).

The expansion and contraction of gene families encoding potential virulence factors are known to be associated with the host range of *Colletotrichum* species, but it is not known whether expanded or contracted gene families are the ancestral state. In the *C. acutatum* species complex, lineage‐specific losses of CAZymes and effectors could be associated with a narrower host range, whereas an expansion of these gene families was found in lineages with a broader host range (Baroncelli *et al*., 2016). Similarly, the contraction in gene families encoding candidate effectors, CAZymes and enzymes implicated in secondary metabolism in *C. lentis* is likely associated with a narrow host range restricted to a few species in the Fabeae.

### Genotyping by re‐sequencing reveals 10 core and two minichromosomes

High‐density genetic linkage maps are an ideal tool to understand the structural features of a genome, such as the number of rearrangements within, and general organization of, chromosomes, and can be used to assemble supercontigs or scaffolds into pseudomolecules/chromosomes by ordering and orienting them with the help of anchoring genetic markers. However, for fungal species, this requires a laborious process of developing mapping populations originating from biparental crosses.

Unlike other fungal species, fungi in the genus *Colletotrichum* predominantly reproduce asexually, and sexual reproduction is rare in the field (O'Connell *et al*., 2012). The reference isolate CT‐30 (race 0) used in the study is cross‐fertile with isolate CT‐21 (race 1) in the laboratory on sterile lentil stems (Armstrong‐Cho & Banniza, 2006). We generated ascospore‐derived progeny isolates from an *in vitro*‐induced cross between these two isolates. Ninety‐four of these, together with the parental isolates, were sequenced on an Illumina HiSeq 2000 platform, which yielded 598 130 601 paired‐end 100‐bp shotgun sequencing reads. The reads were aligned against the *C. lentis* genome assembly, which resulted in 535 158 586 uniquely mapped reads (89.47%) and 62 972 015 multimapped and unmapped reads (10.53%). The uniquely mapped reads give a 19.08‐fold genomic coverage of isolates with an average of 5574 569 reads per isolate (Table S10). A total of 73 817 polymorphisms between the parental isolates CT‐30 and CT‐21 were called from the uniquely mapped reads using Bowtie2/SAMtools mpileup. Of these, 15 796 were insertion and deletion (INDEL) polymorphisms and 58 021 were SNPs. SNPs are the most frequent genetic variations and provide unique positional information across the population at single base resolution, and therefore were selected for linkage mapping.

Only 14 132 (24%) SNPs remained after a stringent post‐mapping quality filtering based on an allele coverage of five‐fold or more, < 25% missing allele calls per locus and per isolate (SNP calls missing in 26 isolates or fewer) and 0.33 minor allele frequency. The SNPs were assembled into 931 unique haplotypes consisting of 530 genetic bins within which markers showed no evidence of recombination, and 401 singletons (Table 2). The *χ* ^2^ test revealed that 6.12% (57 SNP markers) of the mapped SNP loci deviated significantly (*P *<* *0.05) from the expected 1 : 1 ratio of parental allele contribution considering the haploid genome of *C. lentis* (Table S11).

**Table 2:** Summary of the *Colletotrichum lentis* genetic linkage map

Linkage groups	Genetic bins	Markers in genetic bins	Singleton markers	Unique haplotypes	Genetic distance (cM)
1	52	895	49	101	194.36
2	73	1672	37	110	181.65
3	67	2046	50	117	180.17
4	51	3739	40	91	179.79
5	47	901	37	84	178.90
6	44	969	36	80	149.68
7	59	776	42	101	149.28
8	50	927	33	83	132.53
9	45	561	41	86	129.81
10	34	510	32	66	122.78
11	5	943	4	9	19.83
12	3	193	0	3	7.06
Total	530	14 132	401	931	1625.84

**Figure 6:**
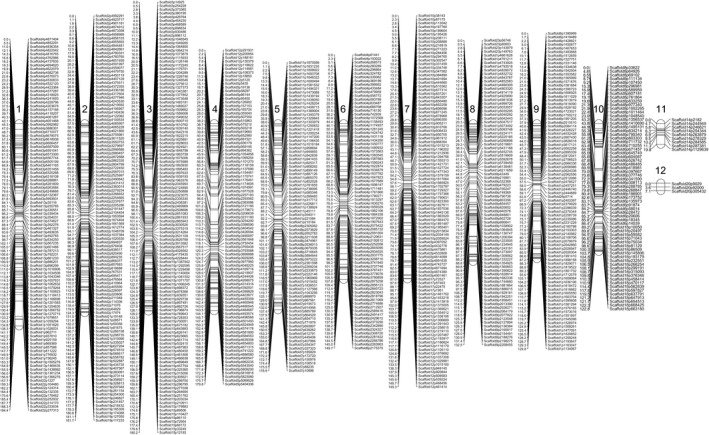
High‐density genetic linkage map of the *Colletotrichum lentis* biparental population (*n *=* *94 ascospore‐derived isolates) originating from a cross between isolates CT‐30 (race 0) and CT‐21 (race 1). To the right of the linkage groups (1–12) are single nucleotide polymorphism (SNP) markers whose genetic positions in centimorgans are shown to the left of the linkage groups.

The high‐quality SNPs were used to construct a linkage map. The SNP markers were mapped into 12 LGs, which is equivalent to the chromosome number of the closely related species *C. higginsianum* (O'Connell *et al*., 2012; Zampounis *et al*., 2016) (Fig. 6). Results of 2D‐checkmatrix (Kozik & Michelmore, 2006) and R/qtl (Broman *et al*., 2003) confirmed the high quality of the genetic linkage map (Fig. S2), which covered a cumulative distance of 1625.8 cM (1 cM = 34 505 bp) with an average intermarker distance of 1.44 cM (49 690 bp). The map covers a similar distance to the amplified fragment length polymorphism (AFLP)‐based map constructed for *C. lindemuthianum* (1897 cM), but is much denser than the average intermarker distance of 11.49 cM for that species (Luna‐Martínez *et al*., 2007). The number of SNP markers varied from three in LG12 to 117 in LG3, and the genetic distance from 7.06 cM (296 404 bp) in LG12 to 194.36 cM (3 785 929 bp) in LG1 (Table 2). The LGs were numbered from LG1 to LG12 by decreasing genetic distance (Fig. 6). LG1 to LG10 spans a higher physical distance (3.70 Mb or more) than the remaining two LGs LG11 (1.13 Mb) and LG12 (0.30 Mb) (Table 3), suggesting that the *C. lentis* genome is organized into 10 core and two minichromosomes. Scaffold14 contains both telomeric ends (CCCTAA)_*n*_ at the 5′‐end and (TTAGGG)_*n*_ at the 3′‐end, and harbors LG11 (1.13 Mb). As a result of low recombination at telomeres, LG11 lacks the ends (telomeres) of scaffold14. Therefore, we conclude that scaffold14 and LG11 both represent the same minichromosome 11 (1.52 Mb). Accumulating evidence suggests that the genomes in the genus *Colletotrichum* are organized into 10 core chromosomes and a variable number of minichromosomes, such as two in *C. higginsianum* (0.65 and 0.61 Mb) and three in *C. graminicola* (ranging from 0.51 to 0.71 Mb) (O'Connell *et al*., 2012).

**Table 3:** Genetic map‐guided *Colletotrichum lentis* genome assembly at the pseudomolecule level

Chromosomes	Size (Mb)	GC (%)	Repeat element kb (%)	Retroelements	DNA transposons	Small RNA
Chr1	3.785929	51.87	129.09 (3.41%)	34	1	0
Chr2	6.04485	52.10	182.09 (3.01%)	46	13	0
Chr3	5.611468	51.19	215.13 (3.83%)	88	17	1
Chr4	5.770251	49.38	231.47 (4.01%)	95	22	0
Chr5	6.258091	49.88	191.86 (3.07%)	64	11	0
Chr6	4.623406	49.55	194.63 (4.21%)	104	21	0
Chr7	4.675034	50.11	190.46 (4.07%)	73	6	1
Chr8	4.085457	50.82	180.75 (4.42%)	92	14	1
Chr9	4.219243	50.94	136.95 (3.25%)	26	5	0
Chr10	3.697061	50.97	143.32 (3.88%)	35	11	2
Chr11	1.128011	38.60	96.78 (8.58%)	102	31	0
Chr12	0.296404	38.60	23.16 (7.81%)	18	15	0
Unassembled	5.904878	47.99	342.54 (5.80%)	239	112	2
Total	56.100083	48.62	2258.23 (4.57%)	1016	279	7

### Genome assembly at the chromosome level and comparative mapping

Twenty‐two scaffolds of the *C. lentis* genome assembly ranging from 0.16 to 6.69 Mb were ordered and oriented into 12 chromosomes using the unique coordinates of the 931 SNP markers distributed on the CT‐30 × CT‐21 linkage map (Table S12). The chromosomes contain 46.83 Mb of the 56.10‐Mb genome assembly. Furthermore, we took advantage of genetic bins to further extend the chromosome lengths as SNP markers within the genetic bins share the same genetic distance (identical haplotypes) whilst differing in physical distance. This resulted in the assignment of an additional 3.38 Mb to chromosomes, increasing the size of sequences assembled into chromosomes to 50.21 Mb (89.51% of the genome), leaving 11.49% (5.90 Mb) of the genome unassembled (Table 3).

**Figure 7:**
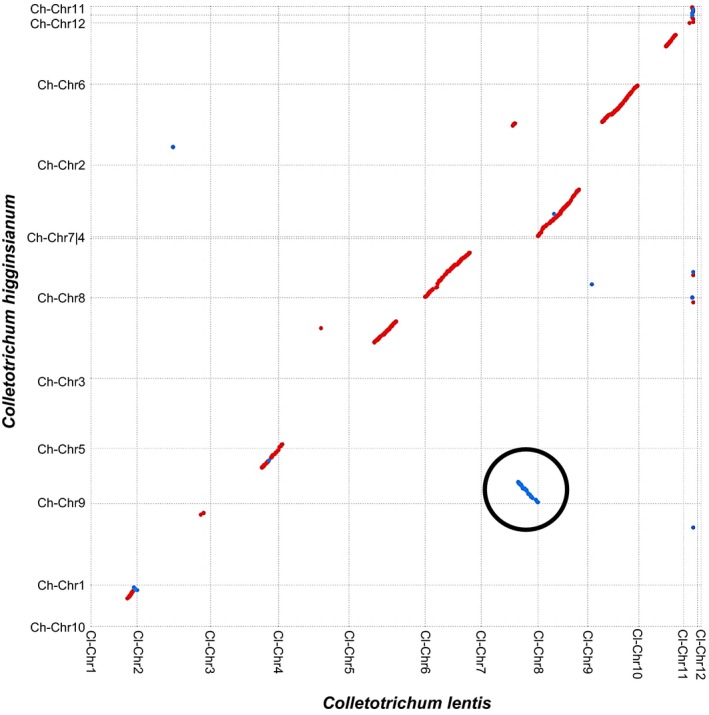
Dot plot representing the correspondence between *Colletotrichum higginsianum* chromosomes (Ch‐Chr1 to Ch‐Chr12, *x*‐axis) and *C. lentis* chromosomes (Cl‐Chr1 to Cl‐Chr12, *y*‐axis). Translocation is circled in black. Red dots (lines) represent sequences aligning in the same direction, whereas blue dots (lines) show sequences aligning in the opposite direction.

Comparative analysis reveals a truncated collinearity along the pseudomolecules of *C. lentis* and C. *higginsianum*. A high level of collinearity was identified between *C. lentis* (Cl)‐Chromosome (Chr) 6 and *C. higginsianum* (Ch)‐Chr8, and between Cl‐Chr8 and Ch‐Chr4 (Fig. 7). A reciprocal translocation was observed in *C. lentis* between chromosomes 3 and 7 (Fig. 7). The minichromosome Cl‐Chr11 is syntenic to Ch‐Chr11 and Ch‐Chr12, suggesting that both *C. higginisianum* minichromosomes may be two segments (0.65 and 0.61 Mb) of a larger minichromosome (1.26 Mb) that is almost similar in size to Cl‐Chr11. Both *C. lentis* minichromosomes Cl‐Chr11 and Cl‐Chr12 contain 38.60% GC content, which is significantly lower than that of the core genome (50.68% for Chr1 to Chr10). At the same time, both minichromosomes are enriched with repetitive DNA (averaging 8.2%) compared with the core genome (3.72%) (Table 3). These minichromosomes are gene sparse as the gene density (Chr11, 132 genes; Chr12, 31 genes; 111 genes/Mb on average) is just over one‐half of that of the core genome (204 genes/Mb on average). We also checked the SNP, presence/absence and INDEL variants located on minichromsomes in the *C. lentis* biparental population, confirming that all 94 progeny isolates maintained both minichromosomes, suggesting that *C. lentis* does not lose minichromosomes after meiosis (Tables S13–S16).

### QTL mapping reveals a minichromosome governing the *C. lentis* virulence on lentil

To determine the genetic control of virulence in *C. lentis*, the genotyped ascospore‐derived isolates (*n *=* *94) were phenotyped for virulence, measured as anthracnose severity on the differential *L. culinaris* spp. *culinaris* cv CDC Robin in the glasshouse.

**Figure 8:**
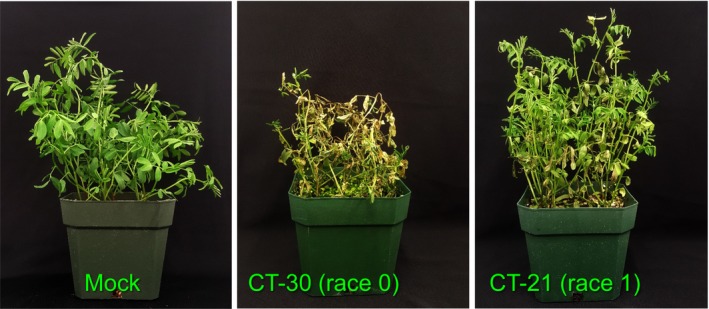
Disease reactions of *Colletotrichum lentis* isolates on the differential *Lens culinaris* ssp. *culinaris* cultivar CDC Robin at 7 d post‐inoculation.

**Figure 9:**
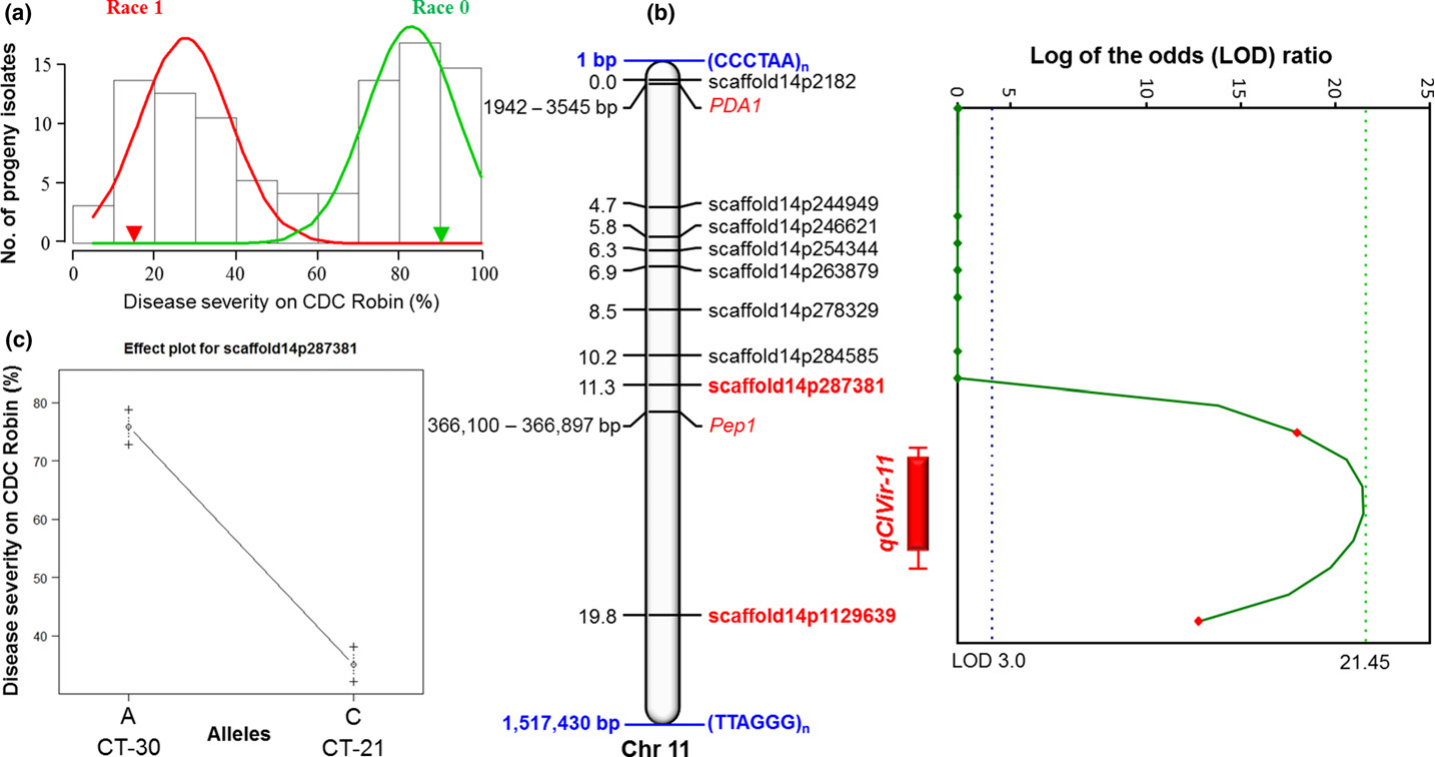
Phenotyping of ascospore‐derived isolates and quantitative trait locus (QTL) mapping. (a) Frequency distribution of anthracnose severity on the differential *Lens culinaris* ssp*. culinaris* cultivar CDC Robin. (b) QTL *qClVIR11* controlling virulence of *Colletotrichum lentis* on lentil is shown as a red solid bar with 1 – LOD confidence intervals. Vertical lines with caps on the bars represent 2 – LOD likelihood intervals. The chart next to the QTL displays the logarithm of the odds (LOD) ratio along minichromosome 11. The chromosome contains telomeric tandem repeats (CCCTAA)_*n*_ at the 5′‐end and (TTAGGG)_*n*_ at the 3′‐end. Blue and green dotted lines represent the LOD score threshold set for QTL detection and the observed LOD score, respectively. (c) Effect plot for the SNP marker scaffold14p287381 linked to the QTL *qClVIR11*.

The disease severity caused by isolates ranged from 9 to 100%. The race 1 isolate CT‐21 caused 16% disease severity on CDC Robin (partially resistant to this race), whereas CT‐30, the race 0 isolate, caused 91% disease severity on CDC Robin, which is susceptible to this race (Fig. 8). Analysis of variance revealed significant effects of isolates on anthracnose severity on CDC Robin (*F* = 14.64, *P *<* *0.001). The frequency distribution of isolates for anthracnose severity on CDC Robin with partial resistance to race 1 revealed a bimodal distribution, indicating that the virulence of *C. lentis* on lentil may be controlled by a single major effect QTL (Fig. 9a).

Composite interval mapping was performed using 931 SNPs and least‐square means of anthracnose severity, resulting in a single QTL *qClVIR11* (LOD = 21.45, *P *<* *0.05) mapped onto the gene‐sparse minichromosome 11 (Figs 9b, S3b, S4). The detection of a single QTL is consistent with the bimodal distribution of anthracnose severity caused by ascospore‐derived isolates on the differential cultivar CDC Robin. The QTL explains 85.23% of the variation in anthracnose severity among isolates, confirming that *qClVIR‐11* is a major effect QTL. The additive genetic effect of the nearest locus, scaffold14p28738, linked to *qClVIR11* was +26.49 (allele A), and therefore, as expected, isolate CT‐30 contributed the virulence allele to the population (Fig. 9c). The QTL flanked by two markers (scaffold14p287381 and scaffold14p1129639) covers a genetic distance of 8.5 cM and a physical distance of 0.84 Mb.

The QTL contains 98 genes, including seven candidate effector and two secondary metabolite genes (Table S17). Two (*PDA1* and *Pep1*) of six pea pathogenicity (*Pep*) cluster gene orthologs were identified on the 1.52‐Mb minichromosome 11. In *Fusarium solani* f. sp. *pisi*, the *Pep* cluster genes are localized in a 1.6‐Mb conditionally dispensable minichromosome, which is essential for its virulence on pea (Fusarium root rot). Four of the *Pep* genes (*PDA1*,* Pep1*,* Pep2* and *Pep5*) are known to contribute to virulence. In *F. oxysporum* f. sp. *pisi*,* PDA1* is always found with *Pep1* on a minichromosome, and both are located in close proximity to each other (Temporini & VanEtten, 2004). For the *F. solani* species complex, it has been speculated that these minichromosomes are acquired by individual isolates through horizontal gene transfer based on the discontinuous phylogenetic distribution of PDA and Pep genes, and that they enable these isolates to colonize additional ecological niches, such as new host species (reviewed in Coleman, 2016). Similar to *F. oxysporum* f. sp. *pisi*,* PDA1* and *Pep1* are 0.36 Mb apart in *C. lentis* (Fig. 9b). Pisatin demethylase encoded by *PDA1* detoxifies the phytoalexin pisatin produced as a defense response by pea (Coleman *et al*., 2011). *PDA1* is located outside of QTL *qClVIR‐11*, whereas *Pep1* is inside the QTL region. The presence of *PDA1* and *Pep1* suggests that minichromosome 11 may be a conditionally dispensable chromosome. It appears that *Pep1* is unlikely to be associated with the difference in virulence between CT‐30 and CT‐21 isolates as the expression of the gene is biologically insignificant (CT‐30/CT‐21 at the biotrophic phase, 5.83 ± 0.41/6.59 ± 0.19 (log_2_ fold ± SEM, *P *>* *0.01); Table S17). Recently, Plaumann *et al*. (2018) have identified two T‐DNA insertion *C. higginsianum* mutants, vir‐49 and vir‐51, defective in the passage from biotrophy to necrotrophy. Both mutants show attenuated virulence on the host *Arabidopsis thaliana* and lack chromosome 11. Similar to our finding in *C. lentis*, the authors concluded that chromosome 11 is a conditionally dispensable minichromosome controlling *C. higginsianum* virulence on *A. thaliana*.

### Concluding remarks

Several genomes in the genus *Colletotrichum* have been sequenced, but only two have been assembled to the chromosome level guided through optical maps (O'Connell *et al*., 2012). Here, we present a population sequencing (POPSEQ)‐based genome assembly of *C. lentis* and provide genetic and physical evidence for the presence of 10 core and two minichromsomes. One of the minichromosomes regulates the *C. lentis* virulence on lentil. To our knowledge, this is the first genetic map‐based genome assembly reported for any fungal plant pathogen.

## Author contributions

S.B. is grant holder. S.B., V.B. and C.P. designed the research and V.B. was the leading researcher. V.B., R.M. and A.C‐S., under the supervision of S.B., performed the experiments and analyzed the data, except for the expression profiling of 22 genes, which was conducted by L.L. and J.H. The research was conducted at the Crop Development Centre/Department of Plant Sciences, University of Saskatchewan, Saskatoon, Saskatchewan, Canada. Genome annotation, masking of repetitive sequences, CAZyme and secondary metabolite identification, prediction of telomeric regions in the scaffolds, and comparative genome mapping and visualization were performed by V.B. at the Swift Current Research and Development Center, Agriculture and Agri‐Food Canada, Swift Current, Saskatchewan, Canada. V.B. drafted the manuscript at the Swift Current Research and Development Center, Agriculture and Agri‐Food Canada, Swift Current, Saskatchewan, Canada with contributions from S.B., who also edited the manuscript. The manuscript was reviewed by all co‐authors.

## Supporting information

Please note: Wiley Blackwell are not responsible for the content or functionality of any Supporting Information supplied by the authors. Any queries (other than missing material) should be directed to the *New Phytologist* Central Office.

**Fig. S1** Comparative analysis of secondary metabolite backbone genes in *Colletotrichum* species.
**Fig. S2** Validation of the genetic linkage map of ascospore‐derived isolates originating from a cross between *Colletotrichum lentis* isolates CT‐30 and CT‐21.
**Fig. S3** Genetic and quantitative trait locus (QTL) mapping.
**Fig. S4** *Colletotrichum lentis* minichromosome 11.
**Table S1** Libraries, sequencing platforms and sequence reads used to generate the *Colletotrichum lentis* genome assembly
**Table S2** General feature format (gff) of *Colletotrichum lentis* protein‐coding gene models
**Table S3** List and functional annotation of candidate effectors predicted in the *Colletotrichum lentis* genome
**Table S4** Log_2_ fold change (log_2_FC) of differentially expressed cysteine‐rich candidate effectors of *Colletotrichum lentis* isolate CT‐30 in susceptible lentil cultivar Eston
**Table S5** Log_2_ fold change (log_2_FC) of differentially expressed candidate effectors of *Colletotrichum lentis* containing < 3% cysteine residues in susceptible lentil cultivar Eston
**Table S6** List and functional annotation of the secondary metabolite backbone genes predicted in the *Colletotrichum lentis* genome
**Table S7** Log_2_ fold change (log_2_FC) of differentially expressed secondary metabolites of *Colletotrichum lentis* backbone genes in susceptible lentil cultivar Eston
**Table S8** List and functional annotation of the carbohydrate‐active enzymes predicted in the *Colletotrichum lentis* genome and associated with the degradation of cell wall components and chitin binding
**Table S9** Log_2_ fold change (log_2_FC) of differentially expressed genes of *Colletotrichum lentis* encoding carbohydrate‐active enzymes in susceptible lentil cultivar Eston
**Table S10** Whole‐genome shotgun sequencing of *Colletotrichum lentis* population
**Table S11** Segregation distortion of single nucleotide polymorphism (SNP) markers
**Table S12** *Colletotrichum lentis* pseudomolecules/chromosomes
**Table S13** qClVir11 haplotype: the qClVir11 region flanked by scaffold14p287381 and scaffold14p1129639 single nucleotide polymorphism (SNP) markers contains 1363 SNPs
**Table S14** Single nucleotide polymorphism (SNP) variants with allele coverage ≥ 5 on minichromosome 12 of *Colletotrichum lentis*
**Table S15** Single nucleotide polymorphism (SNP) variants with allele coverage > 2 on minichromosome 12 of the progeny isolate GT‐2 of *Colletotrichum lentis*
**Table S16** Insertion/deletion (INDEL), presence/absence and polymorphism length on minichromosome 11 of *Colletotrichum lentis*
**Table S17** List and functional annotation of candidate genes underlying the quantitative trait locus (QTL) *qClVIR11* controlling the *Colletotrichum lentis* virulence on lentil, and expression data of CT‐21 and CT‐30 during the biotrophic and necrotrophic phases
**Notes S1** RNA‐sequencing (RNA‐Seq) data analysis.
