# Supplementary material for: Genetic map‐guided genome assembly reveals a virulence‐governing minichromosome in the lentil anthracnose pathogen Colletotrichum lentis
Source: New Phytol. 2018 Aug 4;221(1):431–45. doi: 10.1111/nph.15369 (PMC6668012; doi:10.1111/nph.15369)
Supplement: Supplementary file 1 — Fig. S1 Comparative analysis of secondary metabolite backbone genes in Colletotrichum species. Fig. S2 Validation of the genetic linkage map of ascospore‐derived isolates originating from a cross between Colletotrichum lentis isolates CT‐30 and CT‐21. Fig. S3 Genetic and quantitative trait locus (QTL) mapping. Fig. S4 Colletotrichum lentis minichromosome 11. Table S1 Libraries, sequencing platforms and sequence reads used to generate the Colletotrichum lentis genome assembly Table S2 General feature format (gff) of Colletotrichum lentis protein‐coding gene models Table S3 List and functional annotation of candidate effectors predicted in the Colletotrichum lentis genome Table S4 Log2 fold change (log2FC) of differentially expressed cysteine‐rich candidate effectors of Colletotrichum lentis isolate CT‐30 in susceptible lentil cultivar Eston Table S5 Log2 fold change (log2FC) of differentially expressed candidate effectors of Colletotrichum lentis containing < 3% cysteine residues in susceptible lentil cultivar Eston Table S6 List and functional annotation of the secondary metabolite backbone genes predicted in the Colletotrichum lentis genome Table S7 Log2 fold change (log2FC) of differentially expressed secondary metabolites of Colletotrichum lentis backbone genes in susceptible lentil cultivar Eston Table S8 List and functional annotation of the carbohydrate‐active enzymes predicted in the Colletotrichum lentis genome and associated with the degradation of cell wall components and chitin binding Table S9 Log2 fold change (log2FC) of differentially expressed genes of Colletotrichum lentis encoding carbohydrate‐active enzymes in susceptible lentil cultivar Eston Table S10 Whole‐genome shotgun sequencing of Colletotrichum lentis population Table S11 Segregation distortion of single nucleotide polymorphism (SNP) markers Table S12 Colletotrichum lentis pseudomolecules/chromosomes Table S13 qClVir11 haplotype: the qClVir11 region flanked by scaffold14p287381 and scaffold [file NPH-221-431-s001.pdf]

New Phytologist

***New Phytologist* Supporting Information**

Article title: Genetic map-guided genome assembly reveals a virulence-governing minichromosome in the lentil anthracnose pathogen *Colletotrichum lentis*

Authors: Vijai Bhadauria, Ron MacLachlan, Curtis Pozniak, Aurelie Cohen-Skalie, Li Li, Jerlene Halliday, Sabine Banniza

Article acceptance date: 02 July 2018

The following Supporting Information is available for this article:

<u>Figs S1-S4</u>

Fig. S1 Comparative analysis of secondary metabolite backbone genes in *Colletotrichum* species. PKS, Polyketide synthase; NRPS, Non-ribosomal peptide synthetase; PKS-NRPS, hybrid PKS-NRPS; and DMAT, Dimethylallyl tryptophan synthase.

Fig. S2 Validation of the genetic linkage map of ascospore-derived isolates originated from a cross between *Colletotrichum lentis* isolates CT-30 and CT-21. 2D-CheckMatrix heat plots were generated using the CheckMatrix python script (Kozik & Michelmore, 2006). Each heat plot represents a linkage group. SNP markers are lined up against each other. A red block along the lines indicates tightly linked markers with low recombination. Panels beneath heat maps show the proportion of parental alleles (Red = CT-30, Blue = CT-21). Yellow (CT-30) and light blue (CT-21) colors in the middle represent segregation distortion.

Fig. S3 Genetic and QTL mapping. (a) A side-by-side comparison of genetic maps generated by MadMapper (Kozik & Michelmore, 2006) and R/qtl (Broman *et al.*, 2003). (b) Mapping of QTLs controlling *Colletotrichum lentis* virulence on lentil using R/qtl. Red, blue and green dotted lines represent LOD (log of the odds) score thresholds at 0.1, 0.05 and 0.01 significance levels, respectively.

Fig. S4 *Colletotrichum lentis* minichromosome 11. Bottom, middle and top frames of the figure show GC content, gene density and QTL (*qClVIR11*) position.

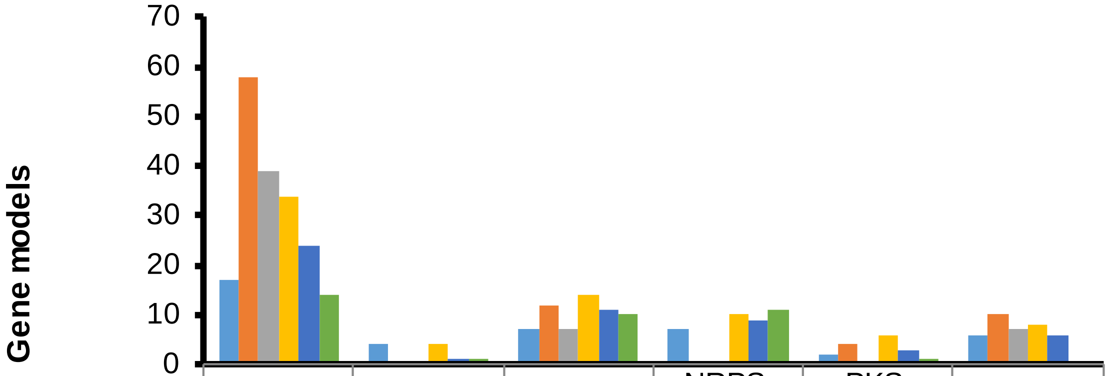

	PKS	PKS-like	NRPS	NRPS-like	PKS-NRPS	DMAT
C. lentis	17	4	7	7	2	6
C. higginsianum	58	0	12	0	4	10
C. graminicola	39	0	7	0	0	7
C. gloeosporiodes	34	4	14	10	6	8
C. orbiculare	24	1	11	9	3	6
F. graminearum	14	1	10	11	1	0

Fig. S1 Comparative analysis of secondary metabolite backbone genes in *Colletotrichum* species. PKS, Polyketide synthase; NRPS, Non-ribosomal peptide synthetase; PKS-NRPS, hybrid PKS-NRPS; and DMAT, Dimethylallyl tryptophan synthase.

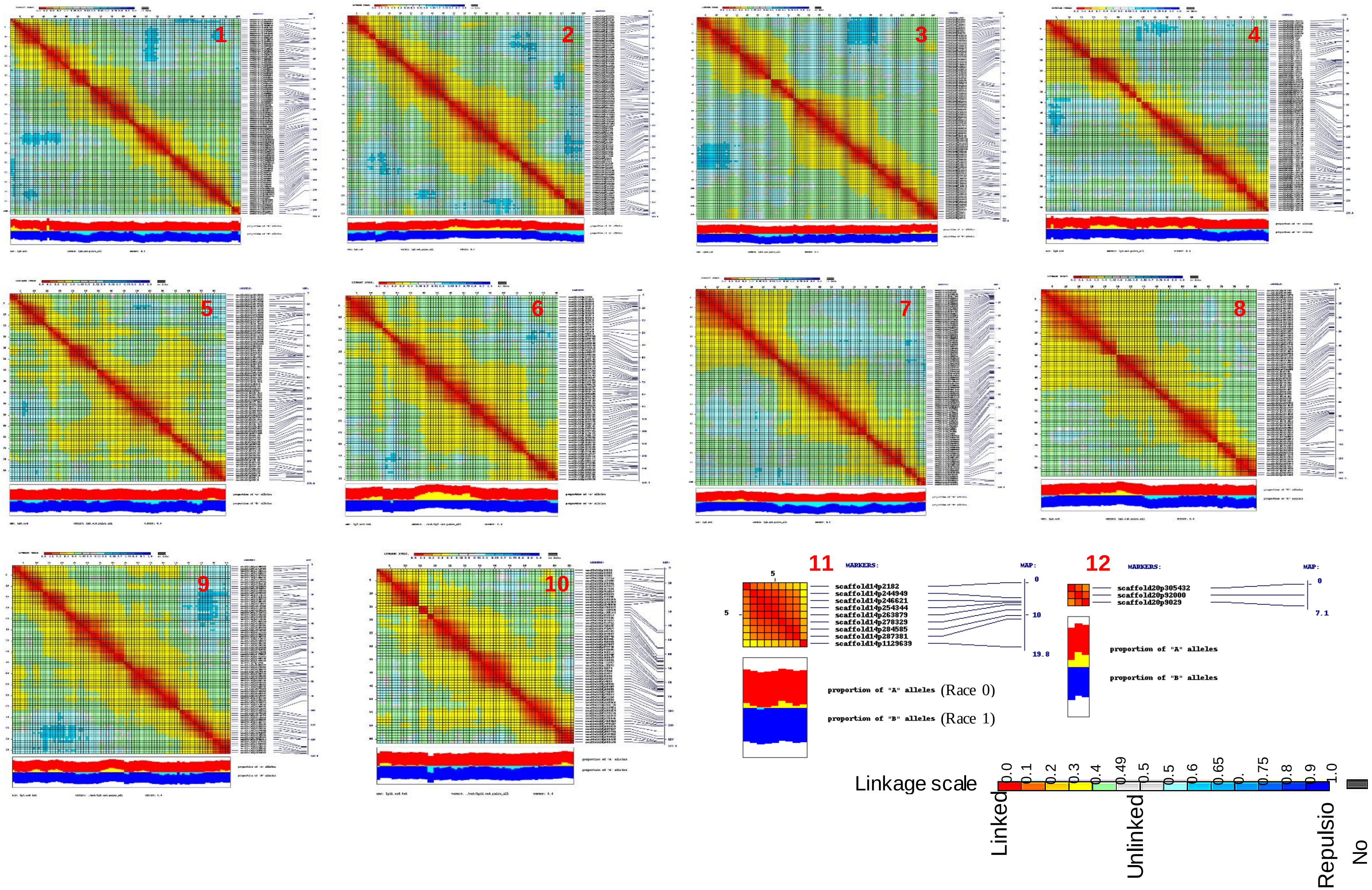

Fig. S2 Validation of the genetic linkage map of ascospore-derived isolates originated from a cross between *Colletotrichum lentis* isolates CT-30 and CT-21. 2D-CheckMatrix heat plots were generated using the CheckMatrix python script (Kozik & Michelmore, 2006). Each heat plot represents a linkage group.

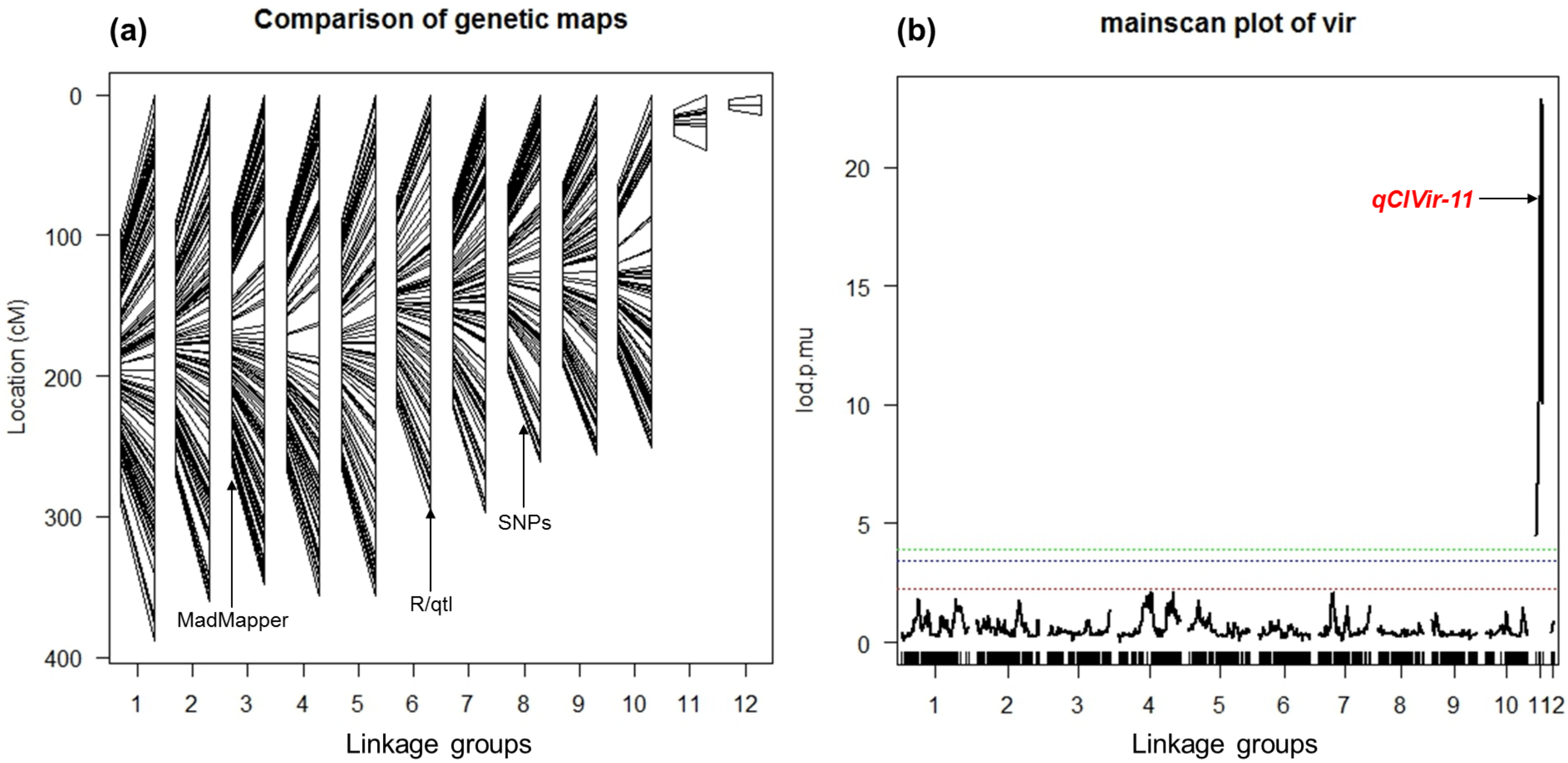

Fig. S3 Genetic and QTL mapping. (a) A side-by-side comparison of genetic maps generated by MadMapper (Kozik & Michelmore, 2006) and R/qtl (Broman *et al.*, 2003). (b) Mapping of QTLs controlling Colletotrichum lentis virulence on lentil using R/qtl. Red, blue and green dotted lines represent LOD (log of the odds) score thresholds at 0.1, 0.05 and 0.01 significance levels, respectively.

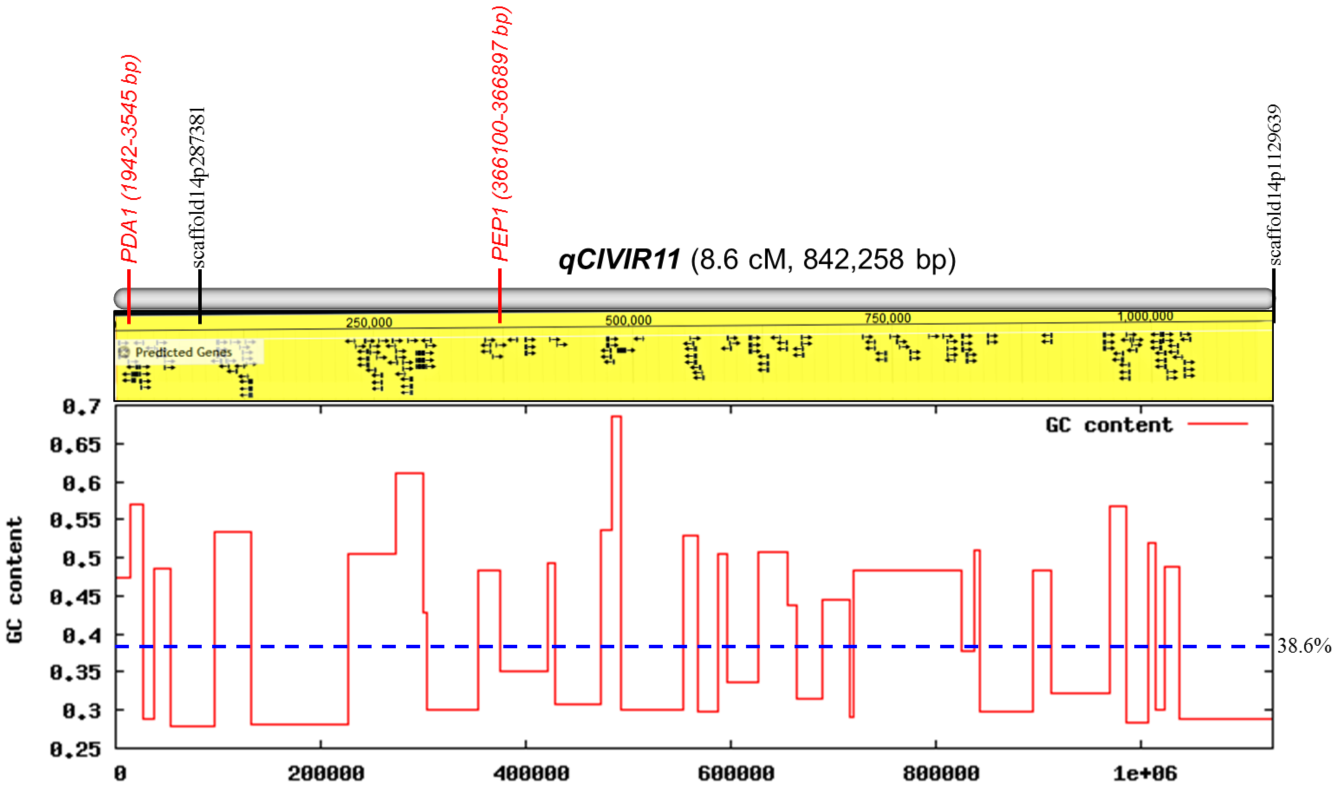

Fig. S4 *Colletotrichum lentis* minichromosome 11. Bottom, middle and top frames of the figure show GC content, gene density and QTL (*qClVIR11*) position.

References

Broman KW, Wu H, Churchill GA. 2003. R/qtl: QTL mapping in experimental crosses. Bioinformatics 19: 889-890.

Kozik A, Michelmore R. 2006. Suite of Python MadMapper scripts for quality control of genetic markers, group analysis and inference of linear order of markers on linkage groups. URL http://cgpdb.ucdavis.edu/XLinkage/MadMapper/. [accessed 11 December 2015].

New Phytologist

New Phytologist **Supporting information**

Article title: Genetic map-guided genome assembly reveals a virulence-governing minichromosome in the lentil anthracnose pathogen Colletotrichum lentis

Authors: Vijai Bhadauria, Ron MacLachlan, Curtis Pozniak, Aurelie Cohen-Skalie, Li Li, Jerlene Halliday, Sabine Banniza

Article acceptance date: 02 July 2018

The following Supporting Information is available for this article:

Notes S1 RNA-Seq data analysis. Below is the entire procedure used to identify differentially expressed genes in *Colletotrichum lentis* isolate CT-30-infected tissues of lentil cultivar Eston.

RNA-Seq data analysis was done following a pipeline described by Haas et al. 2013. The analysis is primarily based on the Trinity assembly and analysis pipeline (Grabherr et al. 2011).

Quality control

Paired-end reads (2x125 bp) were clipped from the 3-prime ends to have a quality score of ≥30 using the Trimmomatic software package (Bolger et al. 2014; Table 1).

Table 1. Read statistic before and after quality control.

Sample[1]	Read set[2]	PE reads[3]	PE reads after quality control[4]	%PE reads after quality control[5]
23hpi-I	23hpi-I.MPS12302102-C10.3648.6	79,716,513	63,890,278	80.1
23hpi-II	23hpi-II.MPS12302102-D10.3648.6	89,351,382	73,282,859	82.0
23hpi-III	23hpi-III.MPS12302102-E10.3648.6	75,714,608	62,948,632	83.1
96hpi-I	96hpi-I.MPS12302102-A11.3648.7	25,540,331	18,973,304	74.3
55hpi-III	55hpi-III.MPS12302102-H10.3648.7	23,319,338	19,528,056	83.7
96hpi-III	96hpi-III.MPS12302102-C11.3648.7	25,076,218	20,130,102	80.3
96hpi-II	96hpi-II.MPS12302102-B11.3648.7	43,514,017	34,341,835	78.9
55hpi-II	55hpi-II.MPS12302102-G10.3648.7	26,456,859	22,114,141	83.6
55hpi-I	55hpi-I.MPS12302102-F10.3648.7	26,695,514	22,235,259	83.3
Myc-II	Myc-II.MPS12302102-A10.3648.7	26,417,771	20,125,499	76.2
Myc-I	Myc-I.MPS12302102-H09.3648.7	24,607,486	19,662,840	79.9
Myc-III	Myc-III.MPS12302102-B10.3648.7	27,578,722	21,169,800	76.8
Myc-I	Myc-I.MPS12302102-H09.3648.8	24,176,050	19,312,394	79.9
Myc-II	Myc-II.MPS12302102-A10.3648.8	25,914,154	19,735,746	76.2
55hpi-I	55hpi-I.MPS12302102-F10.3648.8	26,757,131	22,278,697	83.3
55hpi-II	55hpi-II.MPS12302102-G10.3648.8	28,041,493	23,432,745	83.6
55hpi-III	55hpi-III.MPS12302102-H10.3648.8	24,681,539	20,664,241	83.7
96hpi-II	96hpi-II.MPS12302102-B11.3648.8	43,261,765	34,127,130	78.9
96hpi-I	96hpi-I.MPS12302102-A11.3648.8	27,411,006	20,360,398	74.3

96hpi-III	96hpi-III.MPS12302102-C11.3648.8	25,436,884	20,400,061	80.2
Myc-III	Myc-III.MPS12302102-B10.3648.8	26,949,699	20,670,244	76.7

[1]23 hpi (appressoria-assisted penetration phase), 55 hpi (biotrophic phase) and 96 hpi (necrotrophic phase) and Myc (vegetative mycelia)
[2]Raw 125 bp paired-end reads generated from the Illumina HiSeq platform
[3]Number of paired-end reads survived after quality control
[4]Percentage of survived paired-end reads

De novo transcriptome assembly and annotation

High-quality paired-end reads (phred score ≥30, minimum read length 50) were assembled *de novo* using the Trinity software package (Grabherr et al. 2011; Table 2; Fig. 1). Transcripts were annotated by Trinotate (https://trinotate.github.io/), which uses a number of functional annotation methods, such as homology search using BLAST, SwissProt and Uniref90 databases.

Table 2. De novo transcriptome assembly.

**Features**	**Value**
Nb. Transcripts	216,422
Nb. Components	152,169
Total Transcripts Length (bp)	225,712,952
Min. Transcript Length (bp)	201
Median Transcript Length (bp)	537
Mean Transcript Length (bp)	1,043
Max. Transcript Length (bp)	1,4729
N50 (bp)	1,933

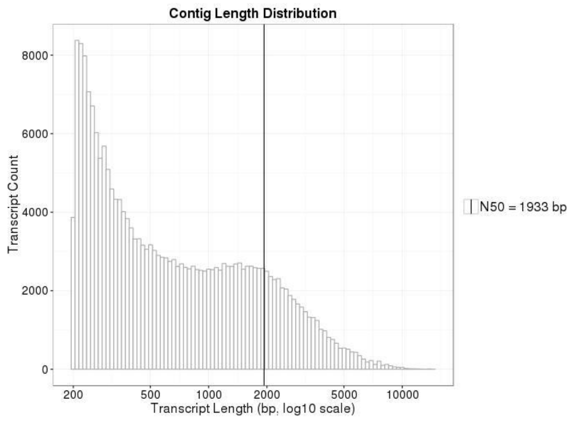

Figure 1. Distribution of transcript length.

Differential gene expression analysis

DESeq (Anders and Huber 2010) and edgeR (Robinson et al. 2010) were used to examine the differential expression of transcripts following the below experimental design (Table 3, Table 4):

Table 3. Experimental design for the expression analysis.

Sample	Myc_vs_23hpi	Myc_vs_55hpi	Myc_vs_96hpi	23hpi_vs_55hpi	23hpi_vs_96hpi	55hpi_vs_96hpi
23hpi-I	2	0	0	1	1	0
23hpi-II	2	0	0	1	1	0
23hpi-III	2	0	0	1	1	0
55hpi-I	0	2	0	2	0	1
55hpi-II	0	2	0	2	0	1
55hpi-III	0	2	0	2	0	1
96hpi-I	0	0	2	0	2	2
96hpi-II	0	0	2	0	2	2
96hpi-III	0	0	2	0	2	2

Myc-I	1	1	1	0	0	0
Myc-II	1	1	1	0	0	0
Myc-III	1	1	1	0	0	0

0, 1 and 2. If a sample is assigned 0, it is not included in a particular analysis. If a sample is assigned 1, the sample is considered as a member of the control group. If a sample is assigned 2, the sample is considered as a member of the test/case group.

Table 4. Snapshot of differentially expressed genes for contrasts.

Differentially Expressed genes for contrast Myc_vs_23hpi							
**id**	**gene_symbol**	**log_FC**	**log_CPM**	**deseq.p-value**	**deseq.adj.pvalue**	**edger.p.value**	**edger.adj.p.value**
TRINITY_DN159362_c0_g1	TRINITY_DN159362_c0_g1	19.038	13.481	0	0	0	0
TRINITY_DN163150_c1_g1	TRINITY_DN163150_c1_g1	19.651	13.633	0	0	0	0
TRINITY_DN175612_c0_g1	TRINITY_DN175612_c0_g1	19.547	13.529	0	0	0	0
TRINITY_DN179977_c0_g1	TRINITY_DN179977_c0_g1	19.748	13.729	0	0	0	0
TRINITY_DN182678_c0_g1	TRINITY_DN182678_c0_g1	19.955	13.936	0	0	0	0
TRINITY_DN184450_c0_g1	TRINITY_DN184450_c0_g1	21.156	13.1	0	0	0	0
TRINITY_DN185405_c0_g1	TRINITY_DN185405_c0_g1	21.693	13.637	0	0	0	0
TRINITY_DN186078_c0_g3	TRINITY_DN186078_c0_g3	22.565	14.509	0	0	0	0
TRINITY_DN191213_c0_g1	TRINITY_DN191213_c0_g1	20.189	14.634	0	0	0	0
TRINITY_DN192168_c0_g1	TRINITY_DN192168_c0_g1	21.283	13.227	0	0	0	0
Differentially Expressed genes for contrast Myc_vs_55hpi							
**id**	**gene_symbol**	**log_FC**	**log_CPM**	**deseq.p-value**	**deseq.adj.pvalue**	**edger.p.value**	**edger.adj.p.value**
TRINITY_DN194141_c0_g1	TRINITY_DN194141_c0_g1	14.908	13.853	0	0	0	0
TRINITY_DN200788_c0_g2	TRINITY_DN200788_c0_g2	15.497	13.418	0	0	0	0
TRINITY_DN203527_c2_g1	TRINITY_DN203527_c2_g1	15.183	13.766	0	0	0	0

New Phytologist

Differentially Expressed genes for contrast Myc_vs_55hpi							
**id**	**gene_symbol**	**log_FC**	**log_CPM**	**deseq.p-value**	**deseq.adj.pvalue**	**edger.p.value**	**edger.adj.p.value**
TRINITY_DN205185_c1_g1	TRINITY_DN205185_c1_g1	15.571	13.921	0	0	0	0
TRINITY_DN194141_c0_g2	TRINITY_DN194141_c0_g2	15.704	12.389	5.5e-255	2.2e-251	0	0
TRINITY_DN201375_c0_g1	TRINITY_DN201375_c0_g1	15.028	12.336	2.7e-251	9e-248	0	0
TRINITY_DN204230_c4_g2	TRINITY_DN204230_c4_g2	14.954	12.274	8.4e-248	2.4e-244	0	0
TRINITY_DN188479_c0_g2	TRINITY_DN188479_c0_g2	15.464	12.146	3.3e-247	8.1e-244	0	0
TRINITY_DN199147_c0_g2	TRINITY_DN199147_c0_g2	14.658	12.204	2e-244	4.4e-241	0	0
TRINITY_DN201190_c0_g1	TRINITY_DN201190_c0_g1	14.465	12.006	3.4e-240	6.9e-237	0	0

Differentially Expressed genes for contrast Myc_vs_96hpi							
**id**	**gene_symbol**	**log_FC**	**log_CPM**	**deseq.p-value**	**deseq.adj.pvalue**	**edger.p.value**	**edger.adj.p.value**
TRINITY_DN10491_c0_g1	TRINITY_DN10491_c0_g1	14.036	8.948	0	0	0	0
TRINITY_DN110236_c0_g1	TRINITY_DN110236_c0_g1	12.965	7.885	0	0	0	0
TRINITY_DN110722_c0_g1	TRINITY_DN110722_c0_g1	10.442	9.049	0	0	0	0
TRINITY_DN115590_c0_g1	TRINITY_DN115590_c0_g1	-6.514	8.709	0	0	0	0
TRINITY_DN14896_c0_g1	TRINITY_DN14896_c0_g1	5.502	9.087	0	0	0	0
TRINITY_DN159438_c0_g1	TRINITY_DN159438_c0_g1	-5.721	9.729	0	0	0	0
TRINITY_DN159559_c0_g2	TRINITY_DN159559_c0_g2	10.795	9.401	0	0	0	0
TRINITY_DN161263_c0_g1	TRINITY_DN161263_c0_g1	11.137	8.852	0	0	0	0
TRINITY_DN162110_c0_g1	TRINITY_DN162110_c0_g1	8.014	8.54	0	0	0	0
TRINITY_DN164844_c0_g1	TRINITY_DN164844_c0_g1	10.06	8.295	0	0	0	0

Differentially Expressed genes for contrast 23hpi_vs_55hpi							
**id**	**gene_symbol**	**log_FC**	**log_CPM**	**deseq.p-value**	**deseq.adj.pvalue**	**edger.p.value**	**edger.adj.p.value**

New Phytologist

Differentially Expressed genes for contrast 23hpi_vs_55hpi							
**id**	**gene_symbol**	**log_FC**	**log_CPM**	**deseq.p-value**	**deseq.adj.pvalue**	**edger.p.value**	**edger.adj.p.value**
TRINITY_DN191680_c0_g2	TRINITY_DN191680_c0_g2	7.501	7.117	0	0	7.2e-302	5.2e-297
TRINITY_DN199868_c2_g1	TRINITY_DN199868_c2_g1	9.054	7.524	0	0	1.5e-279	5.6e-275
TRINITY_DN190033_c0_g1	TRINITY_DN190033_c0_g1	10.233	7.252	6.1e-107	1.4e-103	3.9e-254	9.3e-250
TRINITY_DN230825_c0_g1	TRINITY_DN230825_c0_g1	-7.34	9.217	5.3e-119	1.8e-115	5.9e-248	1.1e-243
TRINITY_DN173194_c0_g1	TRINITY_DN173194_c0_g1	15.382	6.593	0	0	8.3e-245	1.2e-240
TRINITY_DN195988_c0_g2	TRINITY_DN195988_c0_g2	-5.409	11.161	0	0	2.6e-244	3.2e-240
TRINITY_DN173954_c0_g1	TRINITY_DN173954_c0_g1	-7.444	8.994	7.6e-117	2.3e-113	3.3e-238	3.4e-234
TRINITY_DN183140_c0_g2	TRINITY_DN183140_c0_g2	9.941	6.518	5.1e-150	3.4e-146	3.5e-237	3.2e-233
TRINITY_DN35064_c0_g1	TRINITY_DN35064_c0_g1	7.51	6.467	2.3e-79	2.2e-76	2e-226	1.6e-222
TRINITY_DN199220_c0_g3	TRINITY_DN199220_c0_g3	6.89	6.207	7.8e-107	1.7e-103	2e-216	1.5e-212

Differentially Expressed genes for contrast 23hpi_vs_96hpi							
**id**	**gene_symbol**	**log_FC**	**log_CPM**	**deseq.p-value**	**deseq.adj.pvalue**	**edger.p.value**	**edger.adj.p.value**
TRINITY_DN15951_c0_g1	TRINITY_DN15951_c0_g1	9.849	8.99	0	0	0	0
TRINITY_DN182724_c0_g2	TRINITY_DN182724_c0_g2	9.216	8.1	0	0	0	0
TRINITY_DN185504_c0_g1	TRINITY_DN185504_c0_g1	9.174	7.964	0	0	0	0
TRINITY_DN186556_c0_g1	TRINITY_DN186556_c0_g1	8.338	8.993	0	0	0	0
TRINITY_DN187162_c0_g1	TRINITY_DN187162_c0_g1	9.286	7.92	0	0	0	0
TRINITY_DN191663_c0_g1	TRINITY_DN191663_c0_g1	9.165	8.358	0	0	0	0
TRINITY_DN192155_c1_g1	TRINITY_DN192155_c1_g1	18.383	8.943	0	0	0	0
TRINITY_DN201204_c0_g2	TRINITY_DN201204_c0_g2	16.462	11.147	0	0	0	0
TRINITY_DN219047_c0_g1	TRINITY_DN219047_c0_g1	13.189	9.468	0	0	0	0
TRINITY_DN10491_c0_g1	TRINITY_DN10491_c0_g1	18.904	9.467	NA	NA	0	0

Differentially Expressed genes for contrast 55hpi_vs_96hpi							
**id**	**gene_symbol**	**log_FC**	**log_CPM**	**deseq.p-value**	**deseq.adj.pvalue**	**edger.p.value**	**edger.adj.p.value**
TRINITY_DN10491_c0_g1	TRINITY_DN10491_c0_g1	11.862	9.98	0	0	0	0
TRINITY_DN110722_c0_g1	TRINITY_DN110722_c0_g1	13.078	10.082	0	0	0	0
TRINITY_DN14896_c0_g1	TRINITY_DN14896_c0_g1	10.795	10.089	0	0	0	0
TRINITY_DN156182_c0_g2	TRINITY_DN156182_c0_g2	11.265	8.734	0	0	0	0
TRINITY_DN159559_c0_g2	TRINITY_DN159559_c0_g2	12.601	10.436	0	0	0	0
TRINITY_DN161263_c0_g1	TRINITY_DN161263_c0_g1	12.047	9.881	0	0	0	0
TRINITY_DN161449_c0_g1	TRINITY_DN161449_c0_g1	7.954	8.623	0	0	0	0
TRINITY_DN162110_c0_g1	TRINITY_DN162110_c0_g1	11.442	9.563	0	0	0	0
TRINITY_DN171252_c0_g1	TRINITY_DN171252_c0_g1	11.94	10.683	0	0	0	0
TRINITY_DN176858_c0_g1	TRINITY_DN176858_c0_g1	9.424	10.487	0	0	0	0

[1]Gene: component id
[2]Symbol: BLAST protein database id if available, component id otherwise
[3]log_FC: log2 Fold Change of gene level expression
[4]log_CPM: log2 Counts Per Million of gene level expression
[5]deseq.p-value: DESeq nominal p-value
[6]deseq.adj.pvalue: DESeq False Discovery Rate (FDR) adjusted p-value
[7]edger.p-value: edgeR nominal p-value
[8]edger.adj.pvalue: edgeR False Discovery Rate (FDR) adjusted p-value

References

Anders S, Huber W. 2010. Differential Expression Analysis for Sequence Count Data. *Genome Biology* **11**: R106.

Bolger AM, Lohse M, Usadel B. 2014. Trimmomatic: A Flexible Trimmer for Illumina Sequence Data. *Bioinformatics* **30**: 2114-2120.

Grabherr MG, Haas BJ, Yassour M, Levin JZ, Thompson DA, Amit I et al. 2011. Full-length transcriptome assembly from RNA-Seq data without a reference genome. *Nature Biotechnology* **29**: 644-652.

Haas BJ, Papanicolaou A, Yassour M, Grabherr M, Blood PD, Bowden J et al. 2013. De novo transcript sequence reconstruction from RNA-Seq Using the Trinity platform for reference generation and analysis. *Nature Protocols* **8**: 1494-1512.

Robinson MD, McCarthy DJ, Smyth GK. 2010. EdgeR: A bioconductor package for differential expression analysis of digital gene expression data. *Bioinformatics* **26**: 139-140.
